# Uncovering the mouse olfactory long non-coding transcriptome with a novel machine-learning model

**DOI:** 10.1093/dnares/dsz015

**Published:** 2019-07-18

**Authors:** Antonio P Camargo, Thiago S Nakahara, Luiz E R Firmino, Paulo H M Netto, João B P do Nascimento, Elisa R Donnard, Pedro A F Galante, Marcelo F Carazzolle, Bettina Malnic, Fabio Papes

**Affiliations:** 1 Department of Genetics and Evolution, Institute of Biology, University of Campinas, Campinas, SP, Brazil; 2 Graduate Program in Genetics and Molecular Biology, Institute of Biology, University of Campinas, Campinas, SP, Brazil; 3 Department of Biochemistry, Institute of Chemistry, University of Sao Paulo, Sao Paulo, SP, Brazil; 4 Molecular Oncology Center, Hospital Sirio-Libanes, Sao Paulo, SP, Brazil

**Keywords:** long non-coding RNAs, machine learning, olfaction, transcriptome

## Abstract

Very little is known about long non-coding RNAs (lncRNAs) in the mammalian olfactory sensory epithelia. Deciphering the non-coding transcriptome in olfaction is relevant because these RNAs have been shown to play a role in chromatin modification and nuclear architecture reorganization, processes that accompany olfactory differentiation and olfactory receptor gene choice, one of the most poorly understood gene regulatory processes in mammals. In this study, we used a combination of *in silico* and *ex vivo* approaches to uncover a comprehensive catalogue of olfactory lncRNAs and to investigate their expression in the mouse olfactory organs. Initially, we used a novel machine-learning lncRNA classifier to discover hundreds of annotated and unannotated lncRNAs, some of which were predicted to be preferentially expressed in the main olfactory epithelium and the vomeronasal organ, the most important olfactory structures in the mouse. Moreover, we used whole-tissue and single-cell RNA sequencing data to discover lncRNAs expressed in mature sensory neurons of the main epithelium. Candidate lncRNAs were further validated by *in situ* hybridization and RT-PCR, leading to the identification of lncRNAs found throughout the olfactory epithelia, as well as others exquisitely expressed in subsets of mature olfactory neurons or progenitor cells.

## 1. Introduction

In the last decades, several types of long non-coding RNAs (lncRNAs)—transcripts longer than 200 nt characterized by the absence of long open reading frames—have been shown to regulate a variety of biological processes,^1^ but the function of most of them remains enigmatic. Most lncRNAs are transcribed by RNA polymerase II^2^ and exhibit prominent tissue-specific expression.^3^ Even though they share some defining features, their modes of action involve a plethora of distinct molecular mechanisms, including modulation of nuclear architecture, chromatin modification, DNA methylation, transcription regulation, post-transcriptional processing, translation regulation, RNA stability control, biogenesis of miRNAs, and control of protein activity.^4–6^ An example of mammalian lncRNA with known molecular mechanism is *Xist,* a long non-coding transcript that recruits a series of molecular players to initiate large-scale silencing of one X chromosome in female cells.^7^ Most described lncRNAs recruit histone complex modifiers, such as polycomb-repressive complex 2, to initiate remodelling of various portions of the genome.^8^ Other lncRNAs seem to act by modulating DNA methylation, such as a recently described lncRNA that, once transcribed, controls DNA methylation at a nearby promoter in the protocadherin-α gene, leading to increased transcription of the corresponding protein-coding RNA through stochastic alternative promoter usage in sensory neurons of the mouse olfactory system.^9^

Olfaction is probably the most important sense for terrestrial animals,^10^ being crucial for the recognition of a range of environmental stimuli and for appropriate behavioural, physiological, and endocrine responses related to finding food, avoiding danger, and interacting with individuals of the same species. The two most important olfactory sensory organs in mammals are the main olfactory epithelium (MOE) and the vomeronasal organ (VNO), located in the nasal cavity. The MOE is related to the general perception of odours,^10^ while the VNO is typically associated with instinctive behaviours triggered by intra- and inter-specific communication cues, such as sex, aggression, territoriality, parental care, and defensive responses towards predators.^11^

During olfactory sensory neuron differentiation, several molecular mechanisms involving chromatin remodelling take place to shape the detection properties of these cells.^12–15^ For example, each MOE neuron singularly expresses only one type of odorant receptor gene allele chosen among 2,000 possible loci in the genome, a process shown to involve specific epigenetic changes in the genome.^13^^,^^15–20^ Given the role of lncRNAs in chromatin remodelling, we hypothesize they may play a role in controlling how olfactory neurons attain their unique molecular characteristics.

In this article, we used a combination of transcriptome analyses and a machine-learning model to provide for the first time a comprehensive list of lncRNAs in olfaction, including annotated as well as a large set of novel non-coding transcripts. We also identified lncRNAs preferentially expressed in the mouse olfactory organs and determined the temporal patterns of olfactory lncRNA expression during organismic development and sensory neuron differentiation. As an example of how the list of lncRNAs could be useful, we uncovered lncRNAs expressed in mature sensory neurons of the MOE as well as an lncRNA expressed in progenitor cells of the VNO.

## 2. Materials and methods

### 2.1. Transcriptome assembly

We used data from 5 different studies,^21–25^ totalling 49 samples, of which 43 are from adult mice (brain, cerebellum, cerebral cortex, heart, kidney, liver, MOE, and VNO) and 6 are from newborn animals (MOE and VNO). Splicing-aware read mapping was performed using STAR (version 2.5.1b)^26^ with a 2-pass mapping step to gather splice junctions detected during the first mapping step. The transcriptomes were assembled with Cufflinks (version 2.2.1)^27^ to the GRCm38 primary assembly masked version, downloaded from Ensembl, and Cuffmerge was used to merge together the resulting assemblies, using the ‘ref-gtf’ parameter to include GENCODE’s comprehensive gene annotation (release M9).

### 2.2. Candidate lncRNA identification in the assembled transcriptome

To identify possible lncRNAs in the transcriptome reconstructed by Cuffmerge, we adopted a two-step pipeline: firstly, we devised a model of classification of non-coding RNAs using a machine-learning approach with XGBoost (version 0.6 of the xgboost Python package),^28^ and this model was used to identify possible lncRNAs in the transcriptome; next, a genomic overlap filtering step was performed to eliminate non-coding RNAs that do not match the defining criteria for identifying lncRNAs, such as tRNAs, rRNAs, miRNAs, and untranslated region (UTR) fragments. For the latter, we first obtained the sequences of all annotated UTRs in the Ensembl database (release 87) using the biomaRt software (version 2.30).^29^ Next, the length distributions of the sequences obtained were inspected, and we selected their upper quartile [160 bp (5′-UTR) and 792 bp (3′-UTR)]. A GTF file containing intervals corresponding to the annotated coding regions plus the representative length values for the UTRs (artificial coding transcripts) was generated and we excluded one-exon transcripts that showed any overlap with the artificial coding transcripts. For transcripts with multiple exons, we excluded only transcripts whose overlap with the artificial coding transcripts comprised >25% of its length. More details on the machine learning and lncRNA identification via the bioinformatics pipeline can be found in the Supplementary Methods section and in the Supplementary Computational Notebook.

### 2.3. Quantification of transcript expression

Kallisto index was generated from the Cuffmerge assembled transcriptome. Transcript abundances were estimated using the ‘bias’ parameter to correct the quantification for sequence bias,^30^ and the parameter ‘bootstrap-samples’ was used to generate 100 bootstrap samples during the expectation-maximization step. For samples with unpaired reads (single-end), the parameters ‘fragment-length’ and ‘sd’ were set to 200 and 80 bp, respectively.^31^ Between-sample abundance normalization was applied to the raw abundance data (TPM values) using library size factors as computed by sleuth (version 0.30.0).^32^ Gene-level abundance was obtained summing up the normalized abundances of all isoforms. Several subsequent steps in our work used transcript level expression values because of the possibility of different isoforms of the same gene exhibiting different expression patterns among tissues and distinct molecular functions.

### 2.4. Identification of transcripts preferentially or differentially expressed in the olfactory organs

To quantify a transcript’s specificity of expression in a given tissue, we used the tspex Python package to calculate the SPM (specificity measure) metric (see Supplementary Methods for details), using log_2_(abundance + 1) values at both transcript and gene levels. Differential expression tests were performed to identify differentially expressed transcripts between two biological conditions (male vs. female and adult vs. newborn). For this, kallisto expression quantification incorporating bootstrap data was analysed with sleuth (version 0.30.0),^32^ using models with covariates indicating the tissue (MOE or VNO), sex (male or female), and age (adult or newborn). Likelihood ratio tests were performed at the transcript level and differentially expressed transcripts were selected at a 5% false-discovery rate threshold.

### 2.5. Analysis of MOE single-cell RNA-Seq libraries

The analysis of MOE scRNA-Seq data was performed with Monocle (version 2.10.0).^33^ Initially, raw abundance data (TPM) of the 93 scRNA-seq samples were converted to absolute abundance (estimated number of RNA molecules per cell) using the built-in Census algorithm (‘relative2abs’ function).^34^ Using DDRTree, the absolute abundance matrix of transcripts with variable expression was reduced to a two-dimensional space (‘reduceDimension’ function), in which the path of the pseudotime underlying the expression data was laid.^35^ Then, the algorithm assigned the position of each cell in that path, that is, the pseudotime associated with that sample (‘orderCells’ function). As the reconstruction of the route was done in an unsupervised way, we defined which end of the pseudotime corresponds to the beginning (precursor cells) and the end (mature neurons) of neurogenesis, based on the expression of *Ascl1* (precursor cell marker) and *Cnga2* (MOE mature sensory neuron marker). Finally, we performed likelihood ratio tests with the ‘differentialGeneTest’ function to identify transcripts that are differentially expressed between OMP-positive and OMP-negative cells. OMP-positive cells were those in which the absolute abundance of the *Omp* gene was >100. Transcripts were selected at a 5% false-discovery rate threshold and the ones with higher average expression in the OMP-positive cells were chosen for further analysis. Smooth spline curves representing transcript expression dynamics along pseudotime were obtained for the selected transcripts using the ‘genSmoothCurves’ function.

## 3. Results

### 3.1. A large set of unannotated lncRNAs identified by a novel computational pipeline involving machine learning

Our objective was to identify lncRNAs preferentially expressed in the olfactory organs, including novel non-coding RNAs. Therefore, we decided not to restrict our analyses to the mouse genome reference annotation. Instead, we used Cufflinks^27^ to assemble a new transcriptome using RNA sequencing (RNA-Seq) data from a variety of mouse tissues and organs (Supplementary Table S1), including adult whole brain, cerebellum, cerebral cortex, liver, kidney, heart, and the olfactory organs MOE and VNO.^24^ The stability of this dataset was checked by comparing with an independently assembled transcriptome using another transcript assembler, StringTie,^36^ leading up to largely overlapping results (Supplementary Fig. S1). The assembled transcriptome contained 226,171 transcripts, distributed in 58,980 loci, of which 15% were not present in the GENCODE reference annotation (release M9).

For this study, we decided to focus on intergenic lncRNAs, the loci of which are not shared with coding genes.^37^ Since many transcripts were not present in the GENCODE annotation, we used a two-step *in silico* strategy to classify RNAs into potentially coding or non-coding species and identify intergenic lncRNAs. This pipeline included (i) a novel machine-learning-based model to determine potentially non-coding transcripts, and (ii) a step to exclude transcripts that overlap with coding genes and genes for other kinds of non-coding RNAs.

**Figure 1:**
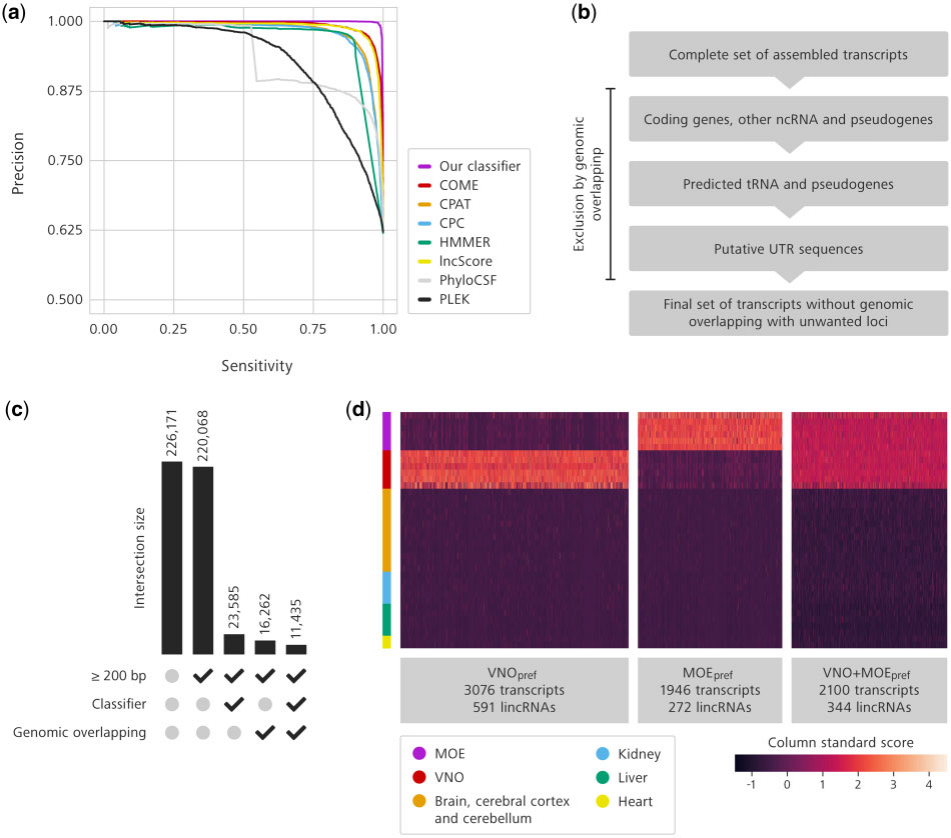
An improved pipeline identifies hundreds of long non-coding RNAs preferentially expressed in the olfactory organs. (a) Precision-recall curve of our lncRNA classifier (purple) in comparison with other currently available classification models. (b) Schematic representation of the sequential removal of transcripts displaying genomic overlap with unwanted genomic loci. (c) Bar plot representing the filtering steps for lncRNA identification. Each bar represents the number of transcripts kept by the combination of sieves represented by the check marks below (selection of long transcripts, selection of transcripts that pass our classifier, and selection of transcripts that do not overlap with unwanted genomic loci). The numbers of filtered transcripts are shown above each bar. (d) Heatmap of expression values for lncRNAs preferentially expressed in the MOE (MOE_pref_), in the VNO (VNO_pref_), or in both olfactory organs (VNO+MOE_pref_). Abundance values were normalized by standardized score calculation, which represents deviation of expression from the average. Columns are transcripts and lines are distinct RNA-Seq libraries from tissues represented by the colour code on the left.

We created a new non-coding RNA classification model, which uses a more efficient machine-learning algorithm and a larger set of informative features than currently available lncRNA predictors.^38–42^ It consists of an ensemble of 500 decision trees with a total of 21 features to classify transcripts into coding or non-coding (Supplementary Table S2). We selected transcripts annotated as lncRNA or protein-coding in the GENCODE (release M11) and then randomly assigned them to test or training sets, containing 20 and 80% of the total transcripts, respectively, while maintaining the proportion of coding to non-coding RNAs in each set. The model generated with the training set was used to classify lncRNAs in the test dataset (see Supplementary Methods and Supplementary Computational Notebook for details), and the results were compared with those obtained with other lncRNA prediction software (COME, CPAT, CPC, HMMER,^43^ lncScore, PhyloCSF,^44^ and PLEK) on the same test dataset (Fig. 1a). Our model exhibits a better trade-off between sensitivity and precision, and better overall performance, as summarized by a range of metrics, including accuracy, precision, sensitivity, specificity, and ROC curves (Fig. 1a, Table 1, and Supplementary Fig. S2a).

**Table 1:** Comparison between performance of lncRNA classifiers

Classifier	Accuracy	Sensitivity	Specificity	Precision	Area under the precision-recall curve	Area under the ROC curve
Our classifier	0.9972	0.9898	0.9989	0.9952	0.9996	0.9999
COME	0.9285	0.9639	0.9202	0.7391	0.9965	0.9859
CPAT	0.8661	0.9624	0.8436	0.5905	0.9906	0.9620
CPC	0.7487	0.9865	0.6930	0.4296	0.9895	0.9585
HMMER	—	—	—	—	0.9842	0.9326
lncScore	0.9374	0.9463	0.9353	0.7742	0.9949	0.9808
PhyloCSF	0.6079	0.8675	0.5471	0.4566	0.9689	0.8891
PLEK	0.7120	0.9069	0.6664	0.5446	0.9640	0.8586

For the computation of each performance metric, we considered lncRNAs as the positive class and coding transcripts as the negative class.

Next, we filtered the assembled transcriptome to remove transcripts overlapping with protein-coding genes and pseudogenes, and excluded long non-coding transcripts that represent or share the same genomic locus with other classes of non-coding RNAs, such as rRNA, tRNA, snoRNA, ribozymes, and miRNA (Fig. 1b).

Transcript assembly software typically return fragments of UTRs of mRNAs as independent transcripts,^45^ which can be misclassified as non-coding RNA. As not all coding transcripts in GENCODE have annotated UTR regions, it is possible that fragments of UTRs may have escaped our first step of filtering. Therefore, an additional step was used to remove candidate lncRNAs lying within 160 and 792 bp from the 5′- and 3′-ends of coding sequences (CDS), respectively. These values were determined from the upper quartile value of UTR lengths calculated from annotated RNAs in GENCODE (Supplementary Fig. S2b).

In total, our classification model identified 23,585 non-coding RNAs that are longer than 200 bp (Fig. 1c), mapping onto 19,448 loci. Of these, 11,435 transcripts in 10,158 loci do not overlap with protein-coding genes, pseudogenes, or other non-coding RNA categories (Fig. 1c). Only 12.8% of all loci (1,301/10,158) have been previously annotated as lncRNAs in GENCODE’s M9 release. Additionally, when we mapped the predicted lncRNA loci onto a reference annotation focussed on non-coding transcripts, NONCODE v5, >45% (4,624/10,158) of our classified lncRNAs fall onto previously mapped non-coding loci.

The resulting comprehensive list of candidate intergenic lncRNAs (Supplementary Spreadsheet), which includes transcripts from both unannotated and annotated loci, was chosen for subsequent investigation.

### 3.2. A multitude of lncRNAs is preferentially expressed in the olfactory organs

Usually, lncRNAs exhibit unique patterns of expression, characterized by preferential or specific expression in tissues or cell types.^3^ Because we were interested in identifying lncRNAs potentially involved in the molecular mechanisms of olfaction, we determined the tissue-specificity of expression for the lncRNAs identified in the previous section. We calculated each transcript’s SPM (*specificity measure*), a parameter that measures the specificity of a gene’s expression in a given tissue or organ.^46^ As a means of comparison, we calculated the SPM for genes known to be preferentially expressed in the olfactory organs, such as *Trpc2*^47^ for the VNO, and *Cnga2*^48^ for the MOE, as well as *Omp*,^49^ which is expressed in both olfactory organs (Supplementary Table S3).

We chose to select genes that are either preferentially expressed in the VNO (VNO_pref_ transcripts), or preferentially expressed in the MOE (MOE_pref_ transcripts), or preferentially expressed in both tissues as compared with other organs/tissues (VNO+MOE_pref_ transcripts). Moreover, we selected SPM cut-off values of 0.9, 0.9, and 0.55 for VNO_pref_, MOE_pref_, and VNO+MOE_pref_ transcripts, respectively, based on SPM values for the marker genes mentioned earlier.

This approach led us to identify 3,076 VNO_pref_, 1,946 MOE_pref_, and 2,100 VNO+MOE_pref_ transcripts, of which 591, 272, and 344, respectively, were classified as lncRNAs (Fig. 1d; Supplementary Spreadsheet). Our improved long intergenic non-coding RNA identification pipeline was specifically devised to identify unannotated non-coding transcripts, and, thus, we expected that few of the lncRNAs listed above would have been annotated in the genome. In fact, only 6.6% (39/591) of the VNO_pref_, 4.0% (11/272) of the MOE_pref_, and 7.6% (26/344) of the VNO+MOE_pref_ lncRNAs are annotated in GENCODE’s M9 release (Supplementary Spreadsheet). When the lncRNAs identified by our classifier were compared with the non-coding specific annotation NONCODE (v5), 14.21% (84/591) of the VNO_pref_, 11.76% (32/272) of the MOE_pref_, and 30.81% (106/344) of the VNO+MOE_pref_ lncRNAs correspond to annotated loci (Supplementary Spreadsheet). Even though these numbers are higher than those found with GENCODE, they are still much lower than the percentage of total lncRNAs in NONCODE, probably due to the fact that the olfactory organs possess peculiar molecular characteristics, such that most of the lncRNAs we detected were probably not previously identified in other studies.

Among the lncRNAs identified in our study, most of the annotated genes are either not expressed or expressed at low levels in the olfactory organs (Supplementary Fig. S3). This probably reflects the fact that most of the lowly expressed lncRNAs are expressed at higher levels in other tissues and organs, in the context of which they have been described. Importantly, most of the lncRNAs that are highly expressed in the olfactory organs have not been previously annotated (Supplementary Fig. S3) and represent novel non-coding RNA species.

### 3.3. Spatial expression patterns for olfactory lncRNAs

Next, we analysed the expression of selected candidate lncRNAs by RT-PCR and *in situ* hybridization to confirm the *in silico* expression quantification and to gain further relevant information about their patterns of expression in the olfactory organs. We chose to evaluate several lncRNAs in each of the three preferential expression groups (VNO_pref_, MOE_pref_, or VNO+MOE_pref_). For each group, we selected (i) lncRNAs whose expression falls above the third quartile of TPM (high expression group), (ii) lncRNAs whose expression falls in the interquartile range (medium expression group), and (iii) lncRNAs of low expression that falls below the first quartile (Supplementary Fig. S4), totalling seven genes analysed per tissue preferential group.

Of these, the only selected transcripts that map onto annotated lncRNA loci in GENCODE are one VNO_pref_ lncRNA (*VNO-D*), five VNO+MOE_pref_ lncRNAs (*VNO/MOE-A, VNO/MOE-B, VNO/MOE-D, VNO/MOE-F, VNO/MOE-G*), and one MOE_pref_ lncRNA (*MOE-A*), all of which have no known biological function (Supplementary Spreadsheet). Moreover, all selected transcripts were chosen among lncRNAs with multiple exons, since these tend to be bona fide transcripts and not the result of purposeless run-off transcription or transcript misassembly.^50^

Next, we performed reverse transcription-PCR (RT-PCR) experiments, with the dual purpose of confirming the complete expressed sequences for each candidate lncRNA and the preferential nature of their expression in olfactory organs. We designed specific PCR primers for each lncRNA (Supplementary Table S4), positioning them on exons near the ends of each transcript.

**Figure 2:**
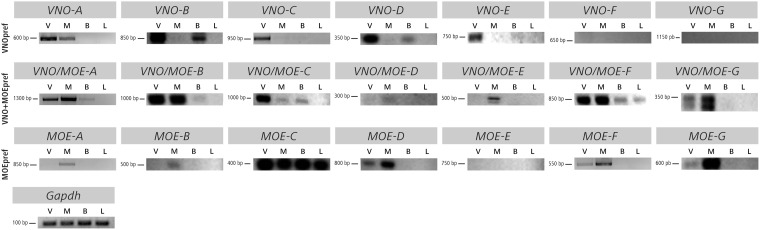
RT-PCR amplification of selected lncRNAs from olfactory organs, brain, and liver tissues. Agarose electrophoresis gels showing amplified bands in RT-PCR experiments for selected lncRNA candidates in the VNO_pref_, VNO+MOE_pref_, and MOE_pref_ preferential expression categories from cDNA prepared from the vomeronasal organ (V), main olfactory epithelium (M), whole brain (B), and liver (L). The first line of images represents investigation of VNO_pref_ lncRNAs, including transcripts in three expression level groups (see Table 2 for details). The middle and bottom lines show transcripts in the VNO+MOE_pref_ and MOE_pref_ categories, respectively. The numbers on the left of each band are the approximate sizes for the amplified bands, which largely overlaps with the *in silico* transcript sequence assemblies (Supplementary FASTA file). Note that *VNO/MOE-G* displays two amplified bands from VNO and MOE, but both were purified and used as probes for *in situ* investigations in Fig. 3. The last image is the endogenous control gene GAPDH, which exhibits unchanged expression levels as judged by band intensity across all samples.

Out of the 16 lncRNA genes in the high and medium expression groups, only one gene, *MOE-E,* could not be amplified by RT-PCR (Fig. 2). The majority of the remaining genes in these expression level categories was found to be expressed in the olfactory organs according to the *in silico* quantifications. For example, *VNO-A* was more highly expressed in the VNO than in the MOE libraries (Table 2), and the RT-PCR data are in agreement with these expression values (Fig. 2). *MOE-C*, which was quantified *in silico* to be more highly expressed in the MOE than in the VNO, was amplified by RT-from both olfactory organs and from other tissues (Fig. 2). Moreover, *VNO/MOE-E*, which exhibits similar abundance levels in the MOE and VNO RNA-Seq libraries, was amplified from MOE tissue. Of the five genes in the low expression category (below the first quartile), two could not be amplified (*VNO-F* and *VNO-G*; Fig. 2) and these have the lowest TPM values (<2.4) across all 21 genes analysed (Table 2).

**Table 2:** Selected lncRNAs preferentially expressed in one or both the olfactory organs

Transcript	Strand	GENCODE M9 annotation	Expression group	VNO		MOE	
				Abundance	SPM	Abundance	SPM
VNO-A	−		High	453.23	0.97	4.87	0.25
VNO-B	+		High	26.22	0.99	0.88	0.14
VNO-C	+		High	7.40	1.00	0.00	0.00
VNO-D	−	Gm33206	Medium	5.00	0.99	0.00	0.00
VNO-E	−		Medium	4.57	1.00	0.06	0.02
VNO-F	−		Low	2.38	1.00	0.00	0.00
VNO-G	+		Low	1.71	1.00	0.00	0.00
MOE-A	n.d.	Gm31557	High	3.67	0.17	17.62	0.98
MOE-B	−		High	1.80	0.11	11.93	0.99
MOE-C	+		Medium	0.29	0.11	7.48	0.94
MOE-D	+		Medium	0.12	0.09	6.46	1.00
MOE-E	+		Medium	0.30	0.18	6.29	0.98
MOE-F	−		Low	0.09	0.11	3.86	0.99
MOE-G	−		Low	0.00	0.00	2.56	1.00
VNO/MOE-A	−	Tmem74bos	High	33.00	0.66	92.17	0.72
VNO/MOE-B	−	BC051077	High	14.55	0.65	35.40	0.68
VNO/MOE-C	+		Medium	8.52	0.76	9.73	0.59
VNO/MOE-D	−	Gm20675	Medium	6.51	0.73	9.00	0.68
VNO/MOE-E	+		Medium	5.04	0.70	5.29	0.56
VNO/MOE-F	+	Gm12996	Medium	4.70	0.70	5.72	0.59
VNO/MOE-G	+	Platr3	Low	2.55	0.79	3.42	0.60

‘Expression group’ refers to the assignments based on expression quartiles for each set of preferentially expressed lncRNAs, according to Supplementary Fig. S4. ‘Abundance data’ means transcripts per million (TPM). n.d., unable to determine expression strand.

All amplified bands were sequenced, revealing experimentally determined transcript sequences that match the *in silico* data (Supplementary FASTA file). Sizes of amplified fragments for these loci were concordant with each amplicon’s expected length, as determined by *in silico* assembly of the corresponding lncRNA transcripts. Exon-intron boundaries were shown to be mostly correct (Supplementary Fig. S5), except in a few cases where the introns were a few nucleotides longer or shorter. The exceptions are: *MOE-D*, which, though amplified as one band, revealed two slightly distinct splicing variants after sequencing; *VNO/MOE-G*, which amplified as two bands; and *MOE-F*, for which the experimentally determined transcript was slightly shorter than the expected size of the lncRNA (Fig. 2), because two exons predicted *in silico* were missing in the PCR amplified band (Supplementary FASTA file).

**Figure 3:**
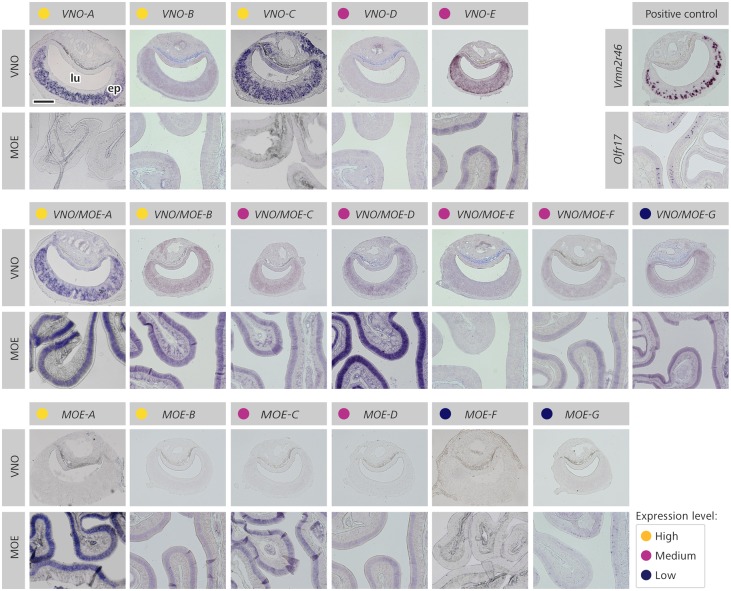
Spatial patterns of expression for selected lncRNAs in the olfactory epithelia of MOE and VNO. Representative microscopy images of olfactory tissue sections subjected to chromogenic *in situ* hybridization staining (purple) with riboprobes for selected preferentially expressed lncRNAs. lncRNA names are as listed on Table 2. The top, middle, and bottom lines of images represent transcripts in the VNO_pref_, VNO+MOE_pref_, and MOE_pref_ groups, respectively. At the end of the first line, controls of hybridization are shown for comparison, including expression of an olfactory receptor gene (*Olfr17*) for the MOE, and staining for vomeronasal organ *Vmn2r46* probe for the VNO. Expression level groups ‘high’, ‘medium’, and ‘low’ were determined according to expression quartiles (Supplementary Fig. S4) and are indicated by yellow, magenta, and blue circles, respectively. lu, VNO lumen; ep, MOE or VNO sensory epithelia. Scale bar = 100 μm.

Next, we conducted *in situ* hybridization for the lncRNA candidates with specific cRNA probes to investigate their spatial patterns of expression in the olfactory organs. For all genes in the high expression category (yellow labels in Fig. 3), we detected clear *in situ* hybridization signal in the sensory epithelium and verified preferential expression data concordant with the *in silico* quantification. For example, *VNO-A* and *MOE-A* exhibit strong expression in the VNO and MOE neuroepithelia, respectively, while *VNO/MOE-A* shows expression in both olfactory organs (Fig. 3; higher magnification images are shown in Supplementary Fig. S6). For lncRNAs in the medium expression group (interquartile TPM range), strong or intermediate staining levels were seen (magenta labels in Fig. 3), except for *VNO-D* in the VNO and for *VNO/MOE-E* in the MOE.

Most lncRNAs with discernible *in situ* hybridization staining in either olfactory organ exhibit a homogeneous spatial pattern of expression in the neuroepithelium (Fig. 3 and Supplementary Fig. S6). Moreover, our data show that most lncRNAs expressed in the MOE are found throughout the neuroepithelium turbinates, without detectable preferential expression in one or more particular dorsal-ventral zones^51^^,^^52^ (Supplementary Fig. S7c and d).

On the other hand, two exceptional lncRNAs exhibit a remarkable punctate staining pattern in the olfactory organs. *VNO-E* seems to be expressed throughout the VNO epithelium, but more highly so in groups of cells lining the base of this olfactory organ and near the epithelial margins (Fig. 3 and Supplementary Fig. S6), the same location of the VNO’s progenitor cells.^53^ Additionally, *MOE-G* is not expressed in the whole MOE, but in a very defined subpopulation of MOE cells (Fig. 3 and Supplementary Figs S6 and S7a and b), which seems to correspond to one of the MOE’s odorant receptor dorsal-ventral expression zones.^51^^,^^52^

For some lncRNA candidates, we performed *in situ* hybridization with probes in the opposite strand, as a control. In the absence of protein-coding information, we synthesized these probes for each lncRNA after confirming the expressed strand with an independent set of MOE RNA-Seq libraries produced via strand-specific SOLiD technology (Table 2). This approach was particularly relevant for intronless transcripts, for which we could not use conserved intron 5′ and 3′ boundary sequences as a proxy to locate the expressed strand. Most of these lncRNA loci showed no significant staining with control probes in the opposite strand, except *VNO/MOE-A_,_* whose expression could be detected with probes in both orientations (Supplementary Fig. S8), suggesting that transcription from this locus is entailed from both strands.

In sum, the novel machine-learning-based pipeline described here allowed us to identify, for the first time, a comprehensive set of long non-coding transcripts in the olfactory organs of mammals. Importantly, we identified hundreds of putative lncRNAs preferentially expressed in the VNO or MOE. We further tested a subset of these transcripts with TPMs encompassing the dynamic range of expression, via a combination of RT-PCR and *in situ* hybridization experiments, which largely confirmed the preferential expression of high- and medium-TPM lncRNAs in either one or both the olfactory organs. Together, these data strongly suggest that the transcripts identified by our bioinformatics pipeline constitute the long non-coding olfactory transcriptome of mice.

### 3.4. Putative olfactory lncRNA loci are not clustered with olfactory receptor genes or enhancers

Loci for all lncRNA transcripts analysed in the previous section were mapped onto the chromosomes in comparison with the location of genes known to be preferentially expressed in the olfactory organs, such as receptor genes in the *Or* (odorant receptor) family expressed in the MOE or in the vomeronasal receptor *V1r* and *V2r* families expressed in the VNO, as well as known olfactory receptor enhancers, such as the H, P, J, and Greek Islands elements, shown to influence the expression of certain groups of odorant receptors in the MOE.^14^^,^^16^^,^^54–59^

**Figure 4:**
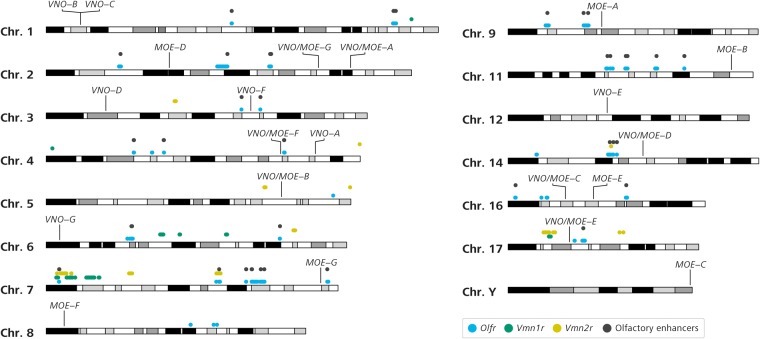
lncRNA loci are not near olfactory enhancers or receptor genes. Chromosomal location of lncRNA loci (named according to Table 2) on the mouse chromosomes, relative to odorant receptor (*Olfr*) gene loci (blue dots), vomeronasal V1R receptor gene (*Vmn1r*) loci (green dots) and vomeronasal V2R receptor gene (*Vmn2r*) loci (yellow dots). Regulatory elements known to be involved in olfactory receptor gene control (P, H, J, and Greek Islands elements) are depicted as grey dots. Only chromosomes with lncRNA loci are shown. Black and greyscale bars represent cytogenetic bands, as retrieved from UCSC’s Genome Browser.

The selected lncRNAs appear not to cluster with those genes and are not in close proximity to the enhancers (Fig. 4). Moreover, when we analysed the distance between all loci for lncRNAs with preferential expression in the olfactory organs and olfactory regulatory sequences, no enhancers were located within 5 kb from the nearest lncRNA gene (not shown).

### 3.5. lncRNA expression in the olfactory organs is not influenced by gender

The VNO is responsible for initiating a series of gender-specific responses, such as aggression between males,^60^^,^^61^ choice of sexual partners,^60^^,^^62^^,^^63^ courtship behaviour, and lordosis behaviour.^64^ Although the MOE is not typically associated with the generation of instinctive behaviours, signalling through this organ is fundamental to the display of dimorphic sexual behaviours in males and females.^65^^,^^66^ Therefore, it is plausible to suspect that the animal’s gender is, to some extent, encoded at the sensory interface of the olfactory organs, in the form of variations in the repertoire of expressed genes, including lncRNAs.

Thus, we conducted *in silico* differential expression tests to investigate whether lncRNA expression is gender-biased or gender-specific. Differential expression analysis between males and females revealed few transcripts differentially expressed between the sexes in the olfactory organs (Supplementary Fig. S9 and Supplementary Table S5). Importantly, none of the differentially expressed genes were lncRNAs. Even though these results are counterintuitive, they are in agreement with another report that did not detect gender-biased expression of olfactory receptors in the MOE or VNO.^24^

### 3.6. A catalogue of lncRNAs expressed in mature olfactory sensory neurons

The differentiation of olfactory neurons is a complex process that involves morphological changes and the acquisition of peculiar molecular characteristics. For example, the differentiating cell undergoes a series of molecular events that culminate with the stochastic choice of one olfactory receptor type to express out of thousands of receptor genes in the genome (singular receptor choice), a phenomenon not completely understood.^67^ It is thought to involve epigenetic modifications known to take place during olfactory differentiation,^68^ as well as nuclear architecture reorganization.^12^^,^^69^ Because lncRNAs have been shown to act through epigenetic mechanisms,^3^ it is conceivable to hypothesize that they play a functional role in one or more of the many cell types along the differentiation lineage, including mature olfactory neurons.

**Figure 5:**
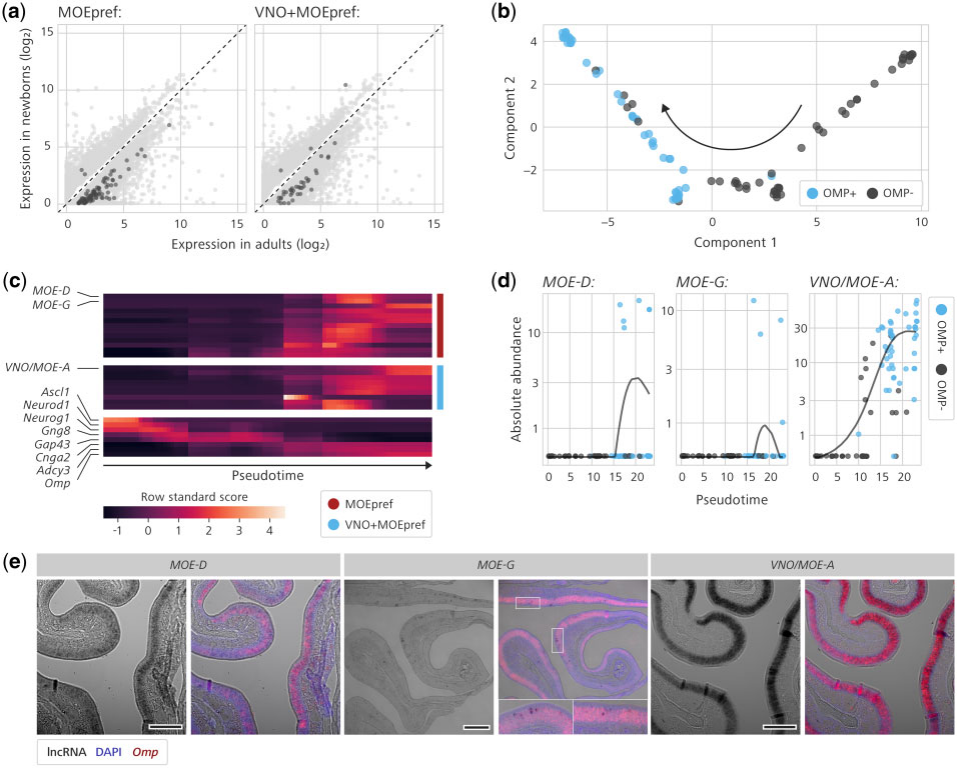
Discovery of lncRNAs differentially expressed in mature olfactory sensory neurons. (a) Mean expression levels (TPM) of all transcripts differentially expressed between adult (x-axis) and newborn (y-axis) in the mouse MOE. Each dot represents a differentially expressed transcript. Dots highlighted in dark grey colour are MOE_pref_ (left) and VNO+MOE_pref_ (right) lncRNA transcripts differentially expressed between adults and newborns. (b) Reconstructed trajectory of cell differentiation in the MOE, represented in a two-dimensional space (constructed using the DDRTree algorithm), based on gene expression analysis of 93 single-cell RNA-Seq transcriptomes.^71^ The black arrow indicates the presumptive progressive temporal sequence of events that transpires from the different single cells along a differentiation pseudotime. Single cells are colour-coded according to presence or absence of OMP expression (cut-off was absolute abundance ≥ 100). (c) Horizontal lines are abundance profiles across the 93 single cells ordered according to the pseudotime determined in (b), for 22 lncRNAs more highly expressed in adults and differentially expressed in OMP-positive single cells (see Supplementary Spreadsheet for complete list). These lncRNAs were taken as putative non-coding species expressed in mature olfactory sensory neurons. Expression of marker genes for several stages along the olfactory differentiation is shown at the bottom. Abundance values were normalized by standardized score calculation. The preferential expression classes (MOE_pref_ or VNO+MOE_pref_) are shown on the right. Three lncRNA candidates are highlighted (*MOE-D*, *MOE-G*, and *VNO/MOE-A*), because they are multi-exon and were chosen for subsequent investigation. (d) Expression abundance data (y-axis) for lncRNAs *MOE-D*, *MOE-G*, and *VNO/MOE-A* along the olfactory differentiation path. The x-axis represents the pseudotime as measured in the bidimensional space. Black and blue dots represent OMP-negative and OMP-positive single cells, respectively. Black lines are smooth spline curves representing transcript expression dynamics along pseudotime, as determined by Monocle 2 software. (e) For each gene (*MOE-D*, *MOE-G*, and *VNO/MOE-A*), the left panel is a lower magnification microscopy black-and-white image of * in situ* hybridization on MOE sections, showing expression of the lncRNA (dark staining) throughout the sensory epithelium (ep) for *MOE-D* and *VNO/MOE-A*, and a striking punctate spatial pattern for *MOE-G*. The right panels are co-labelling for lncRNA (purple) and *OMP* (fluorescent staining in red). DAPI-stained nuclei are shown as blue overlaid fluorescence. For *MOE-G*, higher magnification images of insets in top, right panel are displayed at the bottom, showing details of co-localization of OMP and lncRNA. Size bar is 100 μm.

In order to obtain a comprehensive catalogue of olfactory lncRNAs expressed in mature MOE sensory neurons, we took a two-step approach. First, we reasoned that lncRNAs more highly expressed in adults would be enriched for non-coding transcripts expressed in mature neurons, because the adult epithelium contains a comparatively much larger population of mature neurons than newborns.^70^ The list of transcripts differentially expressed between adults and newborns is extensive (6,784 of 226,171 transcripts) and 67.6% of these are more highly expressed in adults than in newborns (dots below the diagonal 45° dashed line in Fig. 5a). Importantly, we counted 268 lncRNAs (out of 11,435 in total) differentially expressed between adults and newborns, 233 of which (86.9%) are more abundant in the adult MOE.

Considering only the lncRNAs preferentially expressed in the MOE (MOE_pref_), we found 93 lncRNAs (out of 272 in total) that are differentially expressed between adults and newborns (dark dots in Fig. 5a, left), 91 of which are more highly expressed in adults. When we focussed on the lncRNAs preferentially expressed in both olfactory organs relative to other tissues (VNO+MOE_pref_), we found 39 lncRNAs (out of 344 in total) that are differentially expressed between the ages in MOE tissue (dark dots in Fig. 5a, right), 36 of which are prevalently found in adults.

Because we were interested in identifying lncRNAs expressed in mature olfactory neurons, which are positive for Olfactory Marker Protein (OMP) transcripts,^49^ we further filtered the combined list of 91 MOE_pref_ and 36 VNO+MOE_pref_ lncRNA transcripts more highly expressed in adults by selecting those expressed in OMP-positive single MOE cells. These cells are part of a panel of 93 dissociated main olfactory epithelial cells in distinct stages of differentiation.^71^

The dynamics of lncRNA expression in the MOE was analysed using single-cell RNA-Seq (scRNA-Seq) data from the dissociated cells. This approach reconstructed the olfactory lineage differentiation path, as revealed by the position of individual scRNA-Seq samples on a bidimensional space constructed using expression data (Fig. 5b) and comparison with marker genes for several stages along olfactory differentiation, including OMP (Fig. 5c). Of the initial list of 127 (91 + 36) lncRNAs more highly expressed in adults, 9 VNO+MOE_pref_ and 13 MOE_pref_ non-coding transcripts (Supplementary Spreadsheet) were found to be differentially expressed in the OMP-positive subpopulation in comparison with OMP-negative single cells (Fig. 5c), with varying temporal patterns of expression along the differentiation pseudotime.

We chose to validate the expression of multi-exon lncRNAs from this short list of olfactory transcripts expressed in mature sensory neurons. Three transcripts matched these criteria—*VNO/MOE-A*, *MOE-D*, and *MOE-G* (Fig. 5d)—and we performed double *in situ* hybridization experiments with probes for OMP and each of these lncRNAs. Chromogenic detection of hybridized *MOE-G* probe revealed a striking punctate spatial expression pattern in the MOE epithelium (Fig. 3); when combined with fluorescent detection of OMP probe, cells staining positive for the lncRNA clearly co-express the OMP marker (Fig. 5e), indicating that these are mature olfactory neurons and corroborating the *in silico* prediction. Additionally, we investigated lncRNAs *VNO/MOE-A* and *MOE-D* using the same approach: these transcripts are expressed throughout the MOE epithelium (Fig. 3) and we confirmed them to be expressed in mature olfactory neurons by double *in situ* hybridization with OMP (Fig. 5e).

Together, our analyses combined whole tissue and single-cell transcriptome data, differential expression, and *in situ* validation experiments to produce a list of putative lncRNAs expressed in mature olfactory sensory neurons.

### 3.7. One lncRNA is expressed in neural progenitor cells of the VNO

Both the MOE and VNO continuously generate new neurons throughout adult life via differentiation from a stock of progenitor cells located deep within the neuroepithelium.^72^ Olfactory neurogenesis is unique, but few genes have been shown to be specifically expressed in progenitor cells of the olfactory organs. In particular, little is known about the molecular characteristics of the progenitor subpopulation in the VNO.

**Figure 6:**
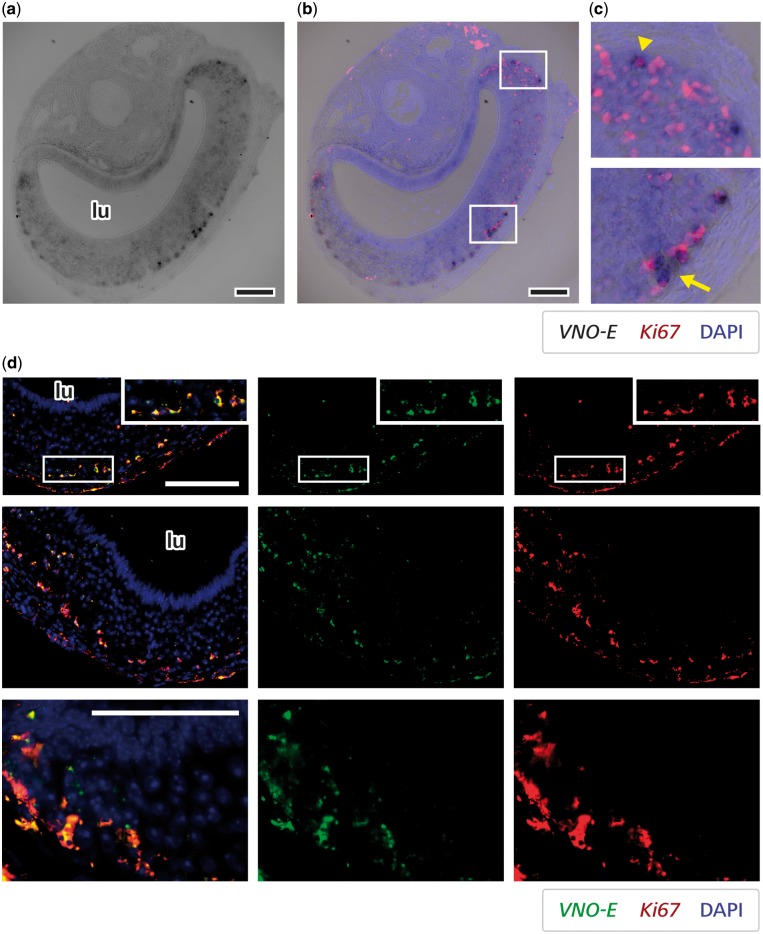
A lncRNA is expressed in vomeronasal progenitor cells. (a) Lower magnification microscopy black-and-white images of VNO sections stained by *in situ* hybridization for lncRNA *VNO-E* probe (dark staining). (b) Co-labelling for *VNO-E* probe (chromogenic detection) and *Ki67* (fluorescent red staining). DAPI-stained nuclei are shown as blue overlaid fluorescence. (c) Higher magnification images of insets in centre panel showing striking localization near the base of the vomeronasal neuroepithelium (arrow) and around the corners (arrowhead). (d) Microscopy images of VNO sections subjected to double fluorescent *in situ* hybridizations for lncRNA *VNO-E* (green) and *Ki67* (red). The first two rows of images depict co-localization between signals for the two genes in cells near the base of the epithelium, confirming the data presented in (a-c). The third row of pictures are high magnification images showing the co-localization of *VNO-E* and *Ki67* in the vast majority of *VNO-E-*positive cells at the corners of the VNO neuroepitelium, where *Ki67* staining is concentrated (neural progenitors). lu, VNO lumen. DAPI is pseudo-coloured in blue. Size bar is 100 μm.

We used *in situ* hybridization to analyse the spatial pattern of expression for one of the lncRNAs discovered in our study, *VNO-E*, which is preferentially expressed in the VNO. Our initial analyses determined that such non-coding transcript is expressed in a small subpopulation of cells near the base and corners of the epithelium (Fig. 3), where progenitor cells are localized. We performed double *in situ* hybridization to investigate whether the *VNO-E* lncRNA is in fact expressed in progenitor cells, using a probe for marker *Ki67*.^73^*Ki67*-positive cells are sparsely distributed along the base and corners of the VNO (Fig. 6), and a fraction of these co-express *VNO-E* overall, suggesting that this lncRNA is expressed in vomeronasal progenitors. Interestingly, it seems that the co-expression levels are more pronounced in *Ki67* cells near the base as compared with the progenitor population at the VNO epithelium corners (Fig. 6), but the functional significance of this apparent difference remains to be determined.

## 4. Discussion

In this study, we used a bioinformatics pipeline containing a novel machine-learning classifier tool to identify long intergenic non-coding RNAs (lncRNAs) in whole-olfactory organ *de novo* transcriptome assemblies. We discovered a range of unannotated lncRNAs preferentially expressed in the olfactory organs, providing the first comprehensive report of lncRNAs in olfaction. We also performed differential expression and single-cell RNA-Seq analyses to gather spatial and temporal expression information. Selected lncRNA candidates were identified in mature olfactory sensory neurons, including one exquisite transcript that exhibits a striking punctate pattern in a subpopulation of these cells. Moreover, we identified transcripts expressed in olfactory progenitor cells. Our work will lay the foundation for future studies on the function of non-coding RNAs in olfaction.

### 4.1. An improved bioinformatics pipeline uncovers olfactory lncRNAs

The lncRNA classification model adopted in our study yields improved classification quality for the identification of lncRNAs over previously published tools (Fig. 1a and Table 1), because it uses a modern machine-learning algorithm and a novel set of features (Supplementary Fig. S10) not used in previously reported prediction algorithms (see Section 2 for details).

Olfactory organs are unique from the molecular and cellular standpoints, and only few studies analysed high-throughput olfactory tissue transcriptomes. Only 1,301 out of the 10,158 lncRNA loci in this study have been annotated in the GENCODE’s M9 release reference mouse genome, and 4,624 have been annotated in the NONCODE v5 version (Supplementary Spreadsheet). Seven lncRNA candidates chosen for further investigation were previously categorized as non-coding transcripts in the GENCODE M9 mouse genome annotation, none of which has assigned biological function.^74^

Our first-hand discovery of lncRNAs in the olfactory organs is relevant, because non-coding transcribed species have been shown to regulate chromatin state, leading to large-scale changes in transcriptional landscape,^75^ processes that take place during olfactory differentiation, for example as part of the regulation of olfactory receptor genes,^12^^,^^17^ one of the molecular hallmarks of sensory neuron differentiation. Therefore, the olfactory lncRNAs we describe here may help the identification of new molecular mechanisms in olfaction.

### 4.2. Preferential expression of lncRNAs in the olfactory organs

The olfactory system displays striking molecular and functional characteristics, including the singular expression of receptor genes, which gives rise to a highly heterogeneous sensory epithelium, in contrast to the much more uniform nature of gene expression in other tissues. Therefore, it makes sense to look for molecular players preferentially or specifically expressed in these sensory structures.

We discovered hundreds of lncRNA candidates preferentially expressed in the olfactory organs (VNO_pref_ or MOE_pref_ groups), or expressed in both organs relative to other mouse tissues (VNO+MOE_pref_). For several selected lncRNA candidates shown *in silico* to be expressed in one or both of the olfactory organs, confirmatory experiments were performed *in situ* (Figs 3, 5e, and 6). Some transcripts exhibited widely distributed expression across the sensory epithelium, whereas another exhibited expression in a restricted subpopulation of mature sensory cells (*MOE-G*), which is reminiscent of the punctate expression pattern of olfactory receptor genes, each of which is expressed in a limited subpopulation of sensory cells widely distributed across the olfactory organ.^76^^,^^77^ Finally, we found one exquisite transcript in the VNO, *VNO-E*, which is expressed in the little understood progenitor cells of that sensory organ.

### 4.3. lncRNA expression in the olfactory organs is influenced by age, but not by gender

The VNO has long been recognized as a sensory organ that chiefly detects pheromones,^11^ substances released by an individual and received by other individuals of the same species, ultimately producing changes in behaviour. Sex pheromones are the most obvious examples, and numerous cases of gender-specific behaviours controlled by pheromones have been described, including the male’s gender discrimination of sexual partners^60^^,^^62^ and female lordosis sexual receptivity behaviour,^64^ to name just a few. Therefore, it seems conceivable that some olfactory genes expressed in the VNO would be gender-specific. However, over the years, it became apparent that gender-biased genes are not the norm.^24^ In agreement with these results, we could not find any lncRNAs differentially expressed between the sexes (Supplementary Fig. S9 and Supplementary Table S5).

On the other hand, we found dozens of lncRNA candidates differentially expressed between adults and newborns (Fig. 5). The presence of age-biased transcripts could be due to several possible reasons, including differences in the cellular composition of the olfactory organs according to age, differences resulting from developmental mechanisms specific to either stage, or functional differences between the olfactory organs in adults and newborns. For example, some behaviours are exhibited differentially according to age, including suckling in juveniles, and nursing, infanticide, pup grooming, intermale aggression, and male and female sexual behaviours.^60–62^^,^^64^^,^^78–82^ It remains to be determined whether the age-biased lncRNAs identified here are involved simply with the control of olfactory organ development or with the regulation of genes related to olfactory stimulus detection and function.

### 4.4. Single-cell RNA-Seq and differential expression analysis reveal lncRNAs expressed in mature olfactory sensory neurons

The olfactory sensory epithelia are exposed to the environment and subject to chemical and biotic injury. To cope with the constant loss of neurons, the olfactory organs harbour permanent pools of stem and progenitor cells, which continuously replenish each organ with newly generated mature olfactory neurons.^83^ In the MOE, progenitor cells give rise to mature olfactory sensory neurons,^72^ and recent single olfactory neuron transcriptome analyses have shed light on the complex dynamics of gene expression along such differentiation process.^71^^,^^84^^,^^85^

We used a combination of bulk RNA-Seq from whole MOE in adults and newborns and single-cell transcriptome analysis to discover 22 olfactory lncRNAs that are at the same time more highly expressed in adults (where the proportion of mature neurons is higher) and in OMP-expressing single cells (which marks mature olfactory neurons) (Fig. 5c). The expression of multi-exon lncRNA candidates fitting these criteria was confirmed in mature olfactory neurons by double *in situ* hybridization with probes for the lncRNA and OMP (Fig. 5e).

Due to the role of lncRNAs in chromatin modification in other cell types,^4^ we hypothesize that these non-coding RNAs play a similar role in olfaction. One possibility is that they may participate in the regulation of olfactory neuron differentiation. Another exciting function for these non-transcribed species is that they may regulate olfactory receptor expression (singular gene choice) or olfactory detection in mature sensory neurons. We anticipate that the comprehensive list of lncRNA candidates and the validated transcripts we provide here will pave the road to better understand the molecular processes of olfaction.

## Ethics approval and consent to participate

Animals used in this study were obtained directly from our vivarium facility, and procedures were carried out in accordance with Animal Protocol no. 1883-1, approved on June 2009 by the Institute of Biology’s Institutional Animal Care and Use Committee (Committee for Ethics in Animal Use in Research), at the University of Campinas, or by animal protocol numbers 19/2013 and 60/2017 approved by the University of São Paulo Chemistry Institute’s IACUC. The protocols follow the guidelines established by the National Council for Animal Experimentation Control (CONCEA-Brazil).

## Supplementary Material

dsz015_Supplementary_Data
