# Supplementary material for: Uncovering the mouse olfactory long non-coding transcriptome with a novel machine-learning model
Source: DNA Res. 2019 Jul 18;26(4):365–78. doi: 10.1093/dnares/dsz015 (PMC6704403; doi:10.1093/dnares/dsz015)
Supplement: dsz015_Supplementary_Data [file dsz015_supplementary_data.zip › dsz015-Suppl_data/Supplementary_computational_notebook.html]

Supplementary computational notebook for "Uncovering the mouse olfactory long non-coding transcriptome with a novel machine-learning algorithm"


# Supplementary computational notebook for "Uncovering the mouse olfactory long non-coding transcriptome with a novel machine-learning algorithm"¶

---

## Computing the features¶

The following software were used to compute the features:

- **COME:** Requires a GTF file (Cufflinks' output) and a pre-built classification model.
- **CPAT:** Requires a FASTA file containing the sequences of the assembled transcriptome, a hexamer-frequency file and a pre-built classification model.
- **CPC:** Requires a FASTA file containing the sequences of the assembled transcriptome and a protein BLAT database. The local database was built using UniRef100 sequences (retrieved in october/2016).
- **HMMER:** Fisrt the transcripts from the Cufflinks' assembled transcriptome were translated in the six reading frames using transeq (from EMBOSS, version 6.6.0). Then, the resulting FASTA file and a protein family HMM file (Pfam, version 30.0) were used as input to the hmmsearch command. Finally, the 'full sequence score' column was extracted to be used as a feature. For the transcripts that had more than one result, the higher value was chosen as a representative. For the transcripts that hadn't appeared in the hmmsearch output, a value of 0 was assigned.
- **lncScore:** Requires a FASTA file containing the sequences of the assembled transcriptome, a hexamer-frequency file and a pre-built classification model.
- **PhyloCSF:** BigWig files containing previously computed PhyloCSF scores (PhyloCSF\_smooth) for each of the six reading frames were retrieved from a online source (https://data.broadinstitute.org/compbio1/PhyloCSFtracks/trackHub/mm10/trackDb.txt). Then, the software bigWigAverageOverBed (version 2.0) was used to calculate the average PhyloCSF score for each one of the six reading frames for each exon and the higher value was chosen as a representative score of the exon. Finally, the transcript's PhyloCSF score was obtained by averaging the exon's scores weighted by the exon's length.

---

In [1]:

```
import warnings
warnings.filterwarnings('ignore')
```

In [2]:

```
import pandas as pd
import numpy as np
import xgboost as xgb
import networkx as nx
from sklearn.model_selection import GridSearchCV, StratifiedKFold
from sklearn.metrics import accuracy_score, recall_score, precision_score, average_precision_score, roc_auc_score, precision_recall_curve, confusion_matrix
import matplotlib.pyplot as plt
import seaborn as sns
sns.set(font='DejaVu Sans', font_scale=1.2, style='whitegrid')
```

---

## Importing the dataset¶

Protein coding and lincRNA transcripts were obtained from the GENCODE M11 comprehensive genome annotation and the softwares listed above were used to calculate a series of features for each transcript.

**Loading the protein coding transcripts dataset:**

In [3]:

```
come_gencode_pc = pd.read_table('Computed_features/COME/come_gencode_pc.out', header=0, index_col=0)
cpat_gencode_pc = pd.read_table('Computed_features/CPAT/cpat_gencode_pc.out', header=0, index_col=0)
cpc_gencode_pc = pd.read_table('Computed_features/CPC/cpc_gencode_pc.out', header=0, index_col=0)
hmmer_gencode_pc = pd.read_table('Computed_features/HMMER/hmmer_gencode_pc.out', header=0, index_col=0)
lncscore_gencode_pc = pd.read_table('Computed_features/lncScore/lncscore_gencode_pc.out', header=0, index_col=0)
phylocsf_gencode_pc = pd.read_table('Computed_features/PhyloCSF/phylocsf_gencode_pc.out', header=0, index_col=0)
```

In [4]:

```
gencode_pc = pd.concat([come_gencode_pc, cpat_gencode_pc, cpc_gencode_pc, hmmer_gencode_pc, lncscore_gencode_pc, phylocsf_gencode_pc], axis=1)
gencode_pc['Labels'] = np.zeros(len(gencode_pc))
```

In [5]:

```
gencode_pc.shape
```

Out[5]:

```
(58429, 34)
```

In [6]:

```
gencode_pc.head()
```

Out[6]:

|  | DNA\_Cons | Protein\_Cons | PolyA- | PolyA+ | smallRNA | GC\_content | H3K36me3 | RNA\_Structure | H3K4me3 | transcript\_size | ... | orfscore | orfdistance | Max\_Mscore\_exon | Max\_distance | Max\_GCcontent | MCS | CSL | CP | phylocsf\_score | Labels |
| --- | --- | --- | --- | --- | --- | --- | --- | --- | --- | --- | --- | --- | --- | --- | --- | --- | --- | --- | --- | --- | --- |
| transcript\_id |  |  |  |  |  |  |  |  |  |  |  |  |  |  |  |  |  |  |  |  |  |
| ENSMUST00000000001.4 | 0.9923 | 0.0 | 0.7178 | 0.9010 | 1.0000 | 0.6944 | 0.7257 | 0.0 | 0.3203 | 3262 | ... | 0.231361 | 0.923559 | 0.331058 | 0.996092 | 0.675676 | 90.775838 | 358.0 | 0.702985 | -2.730284 | 0.0 |
| ENSMUST00000000003.13 | 0.8965 | 0.0 | 0.0000 | 0.0000 | 0.1086 | 0.0833 | 0.1195 | 0.0 | 0.2254 | 902 | ... | 0.081643 | 0.874291 | 0.505110 | 1.389798 | 0.502326 | 26.233620 | 57.0 | 0.608247 | -0.423460 | 0.0 |
| ENSMUST00000000010.8 | 0.9994 | 0.0 | 0.6877 | 0.8957 | 0.2491 | 0.8889 | 0.2882 | 0.0 | 0.3311 | 2574 | ... | 0.368008 | 0.584873 | 0.273626 | 0.340462 | 0.669435 | 94.483135 | 239.0 | 0.706336 | -3.390972 | 0.0 |
| ENSMUST00000000028.13 | 0.9038 | 0.0 | 0.4760 | 0.3003 | 0.6207 | 0.3611 | 0.4001 | 0.0 | 0.4112 | 2143 | ... | 0.341822 | 0.916657 | 0.557984 | 1.589528 | 0.625000 | 214.632589 | 570.0 | 0.906391 | 3.063900 | 0.0 |
| ENSMUST00000000033.11 | 0.9602 | 0.0 | 1.0000 | 0.6179 | 0.3967 | 0.5278 | 0.2334 | 1.0 | 0.6957 | 3708 | ... | 0.328869 | 0.363080 | 0.425545 | 0.454503 | 0.651007 | 67.024786 | 192.0 | 0.704741 | -4.537770 | 0.0 |

5 rows × 34 columns

**Loading the lincRNA dataset:**

In [7]:

```
come_gencode_lnc = pd.read_table('Computed_features/COME/come_gencode_lnc.out', header=0, index_col=0)
cpat_gencode_lnc = pd.read_table('Computed_features/CPAT/cpat_gencode_lnc.out', header=0, index_col=0)
cpc_gencode_lnc = pd.read_table('Computed_features/CPC/cpc_gencode_lnc.out', header=0, index_col=0)
hmmer_gencode_lnc = pd.read_table('Computed_features/HMMER/hmmer_gencode_lnc.out', header=0, index_col=0)
lncscore_gencode_lnc = pd.read_table('Computed_features/lncScore/lncscore_gencode_lnc.out', header=0, index_col=0)
phylocsf_gencode_lnc = pd.read_table('Computed_features/PhyloCSF/phylocsf_gencode_lnc.out', header=0, index_col=0)
```

In [8]:

```
gencode_lnc = pd.concat([come_gencode_lnc, cpat_gencode_lnc, cpc_gencode_lnc, hmmer_gencode_lnc, lncscore_gencode_lnc, phylocsf_gencode_lnc], axis=1)
gencode_lnc['Labels'] = np.ones(len(gencode_lnc))
```

In [9]:

```
gencode_lnc.shape
```

Out[9]:

```
(13695, 34)
```

In [10]:

```
gencode_lnc.head()
```

Out[10]:

|  | DNA\_Cons | Protein\_Cons | PolyA- | PolyA+ | smallRNA | GC\_content | H3K36me3 | RNA\_Structure | H3K4me3 | transcript\_size | ... | orfscore | orfdistance | Max\_Mscore\_exon | Max\_distance | Max\_GCcontent | MCS | CSL | CP | phylocsf\_score | Labels |
| --- | --- | --- | --- | --- | --- | --- | --- | --- | --- | --- | --- | --- | --- | --- | --- | --- | --- | --- | --- | --- | --- |
| transcript\_id |  |  |  |  |  |  |  |  |  |  |  |  |  |  |  |  |  |  |  |  |  |
| ENSMUST00000006814.8 | 0.8404 | 0.0 | 0.1301 | 0.0466 | 0.0911 | 0.4857 | 0.8994 | 0.0 | 0.5810 | 1309 | ... | 0.259995 | 0.696236 | 0.357825 | 1.155837 | 0.590062 | 119.379267 | 412.0 | 0.856437 | 1.539164 | 1.0 |
| ENSMUST00000008011.7 | 0.2981 | 0.0 | 0.0115 | 0.0022 | 0.0237 | 0.3143 | 0.2088 | 1.0 | 0.0919 | 1025 | ... | -0.015150 | 0.512695 | 0.073350 | 0.408290 | 0.519417 | 18.192204 | 41.0 | 0.532474 | -5.225148 | 1.0 |
| ENSMUST00000011196.6 | 0.8203 | 0.0 | 0.0096 | 0.0144 | 0.0264 | 0.9714 | 0.0245 | 0.0 | 0.6108 | 421 | ... | -0.158719 | 0.080838 | 0.080071 | 0.338765 | 0.653846 | 15.724195 | 54.0 | 0.473025 | -0.986874 | 1.0 |
| ENSMUST00000013706.8 | 0.3421 | 0.0 | 0.0053 | 0.0107 | 0.0201 | 0.4286 | 0.2810 | 0.0 | 0.0461 | 1091 | ... | -0.055443 | 0.063441 | -0.084665 | 0.280264 | 0.530726 | 11.809409 | 73.0 | 0.493777 | -2.432992 | 1.0 |
| ENSMUST00000015595.12 | 0.8844 | 0.0 | 0.0174 | 0.0906 | 0.9869 | 0.5714 | 0.5460 | 0.0 | 0.0899 | 5357 | ... | 0.320529 | 0.758382 | 0.528921 | 1.264164 | 0.626374 | 623.523493 | 1735.0 | 0.971185 | 3.152629 | 1.0 |

5 rows × 34 columns

**Merging the datasets into a single DataFrame:**

In [11]:

```
dataset = pd.concat([gencode_pc, gencode_lnc], axis=0)
```

In [12]:

```
X = dataset.drop('Labels', axis=1)
y = dataset['Labels']
```

In [13]:

```
y.value_counts()
```

Out[13]:

```
0.0    58429
1.0    13695
Name: Labels, dtype: int64
```

**Removing duplicated features:**

The feature 'fickett\_score' is calculated by two distinct softwares (CPAT and lncScore) so it's columns is duplicated in the dataframe.

In [14]:

```
print(f'Duplicated columns: {list(X.columns[X.columns.duplicated()])}')
```

```
Duplicated columns: ['fickett_score']
```

In [15]:

```
X = dataset.loc[:, np.invert(X.columns.duplicated())]
```

**Splitting the dataset:**

The transcripts were previously distributed among two sets, the test and the training set, that contain 20% and 80% of the total transcript dataset, respectively. The proportion of coding and non-coding transcripts was kept the same in both sets.

In [16]:

```
with open('Transcript_lists/80_gencode.list') as train_file:
    train_list = [i.strip() for i in train_file]
with open('Transcript_lists/20_gencode.list') as test_file:
    test_list = [i.strip() for i in test_file]
```

In [17]:

```
X_train = X.filter(train_list, axis=0)
X_test = X.filter(test_list, axis=0)
y_train = y.filter(train_list, axis=0)
y_test = y.filter(test_list, axis=0)
```

In [18]:

```
y_train.shape
```

Out[18]:

```
(57699,)
```

In [19]:

```
y_test.shape
```

Out[19]:

```
(14425,)
```

---

## Feature selection¶

In order to avoid overfitting and keep the classification model as simple as possible while mantaining good classification quality, a set of feature selection steps were performed to remove uninformative features.

**Removing zero-variance features:**

In [20]:

```
feature_variance = pd.DataFrame.var(X_train, axis=0)
```

In [21]:

```
print(f'Zero-variance features: {list(feature_variance.index[feature_variance==0])}')
```

```
Zero-variance features: ['Protein_Cons']
```

In [22]:

```
for feature in feature_variance.index:
    if feature_variance[feature] == 0:
        X_train = X_train.drop(feature, axis=1)
        X_test = X_test.drop(feature, axis=1)
```

**Removing unimportant features to the classification model:**

The XGBoost algorithm generates a tree ensemble model in which the feature importance can be quantified in terms of gain, that is, the improvement in accuracy brought by the feature to the branches it is on. In order to simplify the model, features that are unimportant to the model can be removed from the dataset.

In [23]:

```
xgb_feature_model = xgb.XGBClassifier()
xgb_feature_model.fit(X_train, y_train)
```

Out[23]:

```
XGBClassifier(base_score=0.5, booster='gbtree', colsample_bylevel=1,
       colsample_bytree=1, gamma=0, learning_rate=0.1, max_delta_step=0,
       max_depth=3, min_child_weight=1, missing=None, n_estimators=100,
       n_jobs=1, nthread=None, objective='binary:logistic', random_state=0,
       reg_alpha=0, reg_lambda=1, scale_pos_weight=1, seed=None,
       silent=True, subsample=1)
```

In [24]:

```
feature_importance = pd.Series(xgb_feature_model.get_booster().get_score(importance_type='gain')).sort_values(ascending=False)
unimportant_features = list(set(X_train.columns)-set(feature_importance.index))
```

In [25]:

```
print(f'Unimportant features: {unimportant_features}')
```

```
Unimportant features: ['CDS_size', 'RNA_Structure', 'no_hits', 'hit_score', 'orf_integrity', 'frame_score']
```

In [26]:

```
for feature in unimportant_features:
    X_train = X_train.drop(feature, axis=1)
    X_test = X_test.drop(feature, axis=1)
```

**Removing highly correlated features:**

Pairs of highly correlated features contain redundant information concerning the data. Hence, only one feature of each pair of highly correlated features must be kept in the dataset.

In [27]:

```
X_corr = pd.DataFrame.corr(X_train)
X_high_corr = (np.abs(X_corr)>=0.95)
```

In [28]:

```
fig, (ax1, ax2) = plt.subplots(figsize=(14, 10), ncols=2, constrained_layout=True)
sns.heatmap(X_corr, cmap='rocket', square=True, cbar=False, ax=ax1)
ax1.set_title('Correlation between features', fontsize=16, y=1.03)
sns.heatmap(X_high_corr, cmap='rocket', square=True, cbar=False, ax=ax2)
ax2.set_title('Highly correlated features (|correlation| ≥ 0.95)', fontsize=16, y=1.03);
```

In [29]:

```
high_corr_dict = {}
for i in range(len(X_corr.index)):
    for j in range(len(X_corr.columns)):
        if np.abs(X_corr.iloc[j].iloc[i]) >= 0.95:
            if X_corr.index[i] != X_corr.columns[j]:
                if X_corr.index[i] not in high_corr_dict.keys():
                    high_corr_dict[X_corr.index[i]] = [X_corr.columns[j]]
                else:
                    high_corr_dict[X_corr.index[i]].append(X_corr.columns[j])
```

In [30]:

```
for i in high_corr_dict.keys():
    print(f'{i}: {high_corr_dict[i]}')
```

```
orf_size: ['cds_length', 'log-odds_score', 'CSL']
hexamer_score: ['orfscore']
cds_length: ['orf_size', 'log-odds_score', 'CSL']
log-odds_score: ['orf_size', 'cds_length', 'CSL']
orfscore: ['hexamer_score']
CSL: ['orf_size', 'cds_length', 'log-odds_score']
```

In [31]:

```
fig, ax = plt.subplots(figsize=(6,3), constrained_layout=True)
high_corr_graph = nx.Graph(high_corr_dict)
nx.draw(high_corr_graph, ax=ax, with_labels=True, node_color='lightgray', edge_color='gray', node_size=500, width=1.5, font_family='DejaVu Sans')
```

In [32]:

```
high_corr_dropped = []
for cluster in nx.connected_components(high_corr_graph):
    for node in cluster:
        if feature_importance[node] != np.max(feature_importance[cluster]):
            X_train = X_train.drop(node, axis=1)
            X_test = X_test.drop(node, axis=1)
            high_corr_dropped.append(node)
```

In [33]:

```
print(f'Dropped highly correlated features: {high_corr_dropped}')
```

```
Dropped highly correlated features: ['orf_size', 'CSL', 'cds_length', 'hexamer_score']
```

**Final feature set:**

In [34]:

```
selected_features = X_train.columns
for i in selected_features:
    print(i)
```

```
DNA_Cons
PolyA-
PolyA+
smallRNA
GC_content
H3K36me3
H3K4me3
transcript_size
fickett_score
log-odds_score
orf_coverage
hmmer_score
orf_ratio
orfscore
orfdistance
Max_Mscore_exon
Max_distance
Max_GCcontent
MCS
CP
phylocsf_score
```

---

## Training the final classification model¶

**Hyperparameter optimization:**

The `GridSearchCV` function automates the hyperparameter optimization process. It performs an exhaustive grid search from a grid of parameter values in order to find the best combination of hyperparameters.

In [35]:

```
parameters = {'max_depth': [3, 4, 5, 6],
              'learning_rate': [0.05, 0.1, 0.2],
              'n_estimators': [100, 300, 500],
              'gamma': [0, 0.1, 0.2]}
```

In [36]:

```
xgb_model = xgb.XGBClassifier()
xgb_model_tuned = GridSearchCV(xgb_model, parameters, cv=StratifiedKFold(n_splits=5))
xgb_model_tuned.fit(X_train, y_train)
```

Out[36]:

```
GridSearchCV(cv=StratifiedKFold(n_splits=5, random_state=None, shuffle=False),
       error_score='raise',
       estimator=XGBClassifier(base_score=0.5, booster='gbtree', colsample_bylevel=1,
       colsample_bytree=1, gamma=0, learning_rate=0.1, max_delta_step=0,
       max_depth=3, min_child_weight=1, missing=None, n_estimators=100,
       n_jobs=1, nthread=None, objective='binary:logistic', random_state=0,
       reg_alpha=0, reg_lambda=1, scale_pos_weight=1, seed=None,
       silent=True, subsample=1),
       fit_params=None, iid=True, n_jobs=1,
       param_grid={'max_depth': [3, 4, 5, 6], 'learning_rate': [0.05, 0.1, 0.2], 'n_estimators': [100, 300, 500], 'gamma': [0, 0.1, 0.2]},
       pre_dispatch='2*n_jobs', refit=True, return_train_score='warn',
       scoring=None, verbose=0)
```

In [37]:

```
print('Chosen hyperparameters:')
for i,j in xgb_model_tuned.best_params_.items():
    print(f'{i}: {j}')
```

```
Chosen hyperparameters:
gamma: 0.2
learning_rate: 0.1
max_depth: 4
n_estimators: 500
```

**Feature importance:**

In [38]:

```
feature_importance = pd.Series(xgb_model_tuned.best_estimator_.get_booster().get_score(importance_type = 'gain')).sort_values(ascending = False)
fig, ax = plt.subplots(figsize=(10,6), constrained_layout=True)
ax.bar(range(len(feature_importance)), feature_importance.values)
ax.set_title('Feature importance', fontsize=16, y=1.03)
ax.set_ylabel('Gain')
ax.set_xticks(range(len(feature_importance)))
ax.set_xticklabels(feature_importance.index, rotation = 'vertical');
```

---

## Model evaluation¶

**Confusion matrix:**

In [39]:

```
y_predicted = xgb_model_tuned.predict(X_test)
y_predicted_score = xgb_model_tuned.predict_proba(X_test)[:,1]
```

In [40]:

```
tn, fp, fn, tp = confusion_matrix(y_test, y_predicted).ravel()
print(f'True positives: {tp}')
print(f'True negatives: {tn}')
print(f'False positives: {fp}')
print(f'False negatives: {fn}')
```

```
True positives: 2711
True negatives: 11673
False positives: 13
False negatives: 28
```

**Classification quality metrics:**

In [41]:

```
print(f'Accuracy: {accuracy_score(y_test, y_predicted):.4f}')
print(f'Sensitivity: {recall_score(y_test, y_predicted, pos_label=1):.4f}')
print(f'Specificity: {recall_score(y_test, y_predicted, pos_label=0):.4f}')
print(f'Precision: {precision_score(y_test, y_predicted):.4f}')
print(f'Area under the precision-recall curve: {average_precision_score(y_test, y_predicted_score):.4f}')
print(f'Area under the ROC curve: {roc_auc_score(y_test, y_predicted_score):.4f}')
```

```
Accuracy: 0.9972
Sensitivity: 0.9898
Specificity: 0.9989
Precision: 0.9952
Area under the precision-recall curve: 0.9996
Area under the ROC curve: 0.9999
```

**Precision-recall curves:**

In [42]:

```
come_predictions = pd.read_table('PC_curve/Predictions/come_gencode_20.out', header=None)
cpat_predictions = pd.read_table('PC_curve/Predictions/cpat_gencode_20.out', header=None)
cpc_predictions = pd.read_table('PC_curve/Predictions/cpc_gencode_20.out', header=None)
hmmer_predictions = pd.read_table('PC_curve/Predictions/hmmer_gencode_20.out', header=None)
lncscore_predictions = pd.read_table('PC_curve/Predictions/lncscore_gencode_20.out', header=None)
phylocsf_predictions = pd.read_table('PC_curve/Predictions/phylocsf_gencode_20.out', header=None)
plek_predictions = pd.read_table('PC_curve/Predictions/plek_gencode_20.out', header=None)
```

In [43]:

```
xgb_model_precision, xgb_model_sensitivity, _ = precision_recall_curve(y_test, y_predicted_score, pos_label=1)
come_precision, come_sensitivity, _ = precision_recall_curve(come_predictions[1], come_predictions[0], pos_label=0)
cpat_precision, cpat_sensitivity, _ = precision_recall_curve(cpat_predictions[1], cpat_predictions[0], pos_label=0)
cpc_precision, cpc_sensitivity, _ = precision_recall_curve(cpc_predictions[1], cpc_predictions[0], pos_label=0)
hmmer_precision, hmmer_sensitivity, _ = precision_recall_curve(hmmer_predictions[1], hmmer_predictions[0], pos_label=0)
lncscore_precision, lncscore_sensitivity, _ = precision_recall_curve(lncscore_predictions[1], lncscore_predictions[0], pos_label=0)
phylocsf_precision, phylocsf_sensitivity, _ = precision_recall_curve(phylocsf_predictions[1], phylocsf_predictions[0], pos_label=0)
plek_precision, plek_sensitivity, _ = precision_recall_curve(plek_predictions[1], plek_predictions[0], pos_label=0)
```

In [44]:

```
fig, ax = plt.subplots(figsize=(8,6), constrained_layout=True)
ax.plot(xgb_model_sensitivity, xgb_model_precision, label='Ad hoc classifier', color = '#70007c')
ax.plot(come_sensitivity, come_precision, label='COME', color = '#f291d9')
ax.plot(cpat_sensitivity, cpat_precision, label='CPAT', color = '#6565db')
ax.plot(cpc_sensitivity, cpc_precision, label='CPC', color = '#ff7f0e')
ax.plot(hmmer_sensitivity, hmmer_precision, label='HMMER', color = '#aa0009')
ax.plot(lncscore_sensitivity, lncscore_precision, label='lncScore', color = '#eae400')
ax.plot(phylocsf_sensitivity, phylocsf_precision, label='PhyloCSF', color = '#333333')
ax.plot(plek_sensitivity, plek_precision, label='PLEK', color = '#00c181')
ax.set_title('Precision-recall curve', fontsize=16, y=1.03)
ax.set_xlabel('Sensitivity')
ax.set_ylabel('Precision')
ax.legend(loc = 'lower right', frameon=True)
ax.set_xticks(np.linspace(0, 1, num = 5))
ax.set_yticks(np.linspace(0.5, 1, num = 5))
ax.set_xlim(-0.030, 1.030)
ax.set_ylim(0.480, 1.020);
```
