# Supplementary material for: Uncovering the mouse olfactory long non-coding transcriptome with a novel machine-learning model
Source: DNA Res. 2019 Jul 18;26(4):365–78. doi: 10.1093/dnares/dsz015 (PMC6704403; doi:10.1093/dnares/dsz015)
Supplement: dsz015_Supplementary_Data [file dsz015_supplementary_data.zip › dsz015-Suppl_data/Supplementary figures tables and methods.pdf]

Supplementary figures, tables and methods for "Uncovering the mouse olfactory long non-coding transcriptome with a novel machine learning model"

Antonio P. Camargo
Thiago S. Nakahara
Luiz E. R. Firmino
Paulo H. M. Netto
Joao B. P. Nascimento
Elisa R. Donnard
Pedro A. F. Galante
Marcelo F. Carazzolle
Bettina Malnic
Fabio Papes

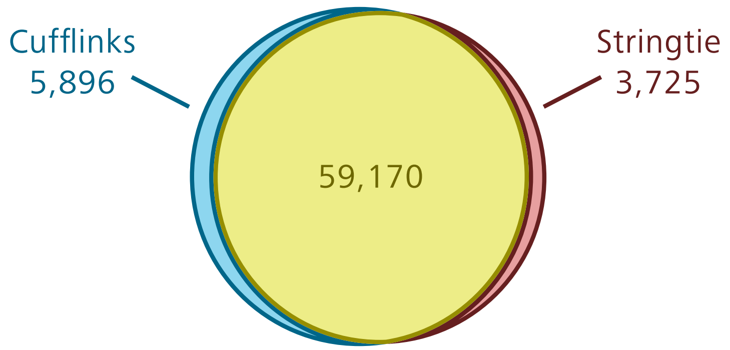

Figure S1. Venn diagram showing that the assemblies of Cufflinks and StringTie are largely concordant. Numbers represent loci longer than 200 bp in each set. Areas are proportional to set size.

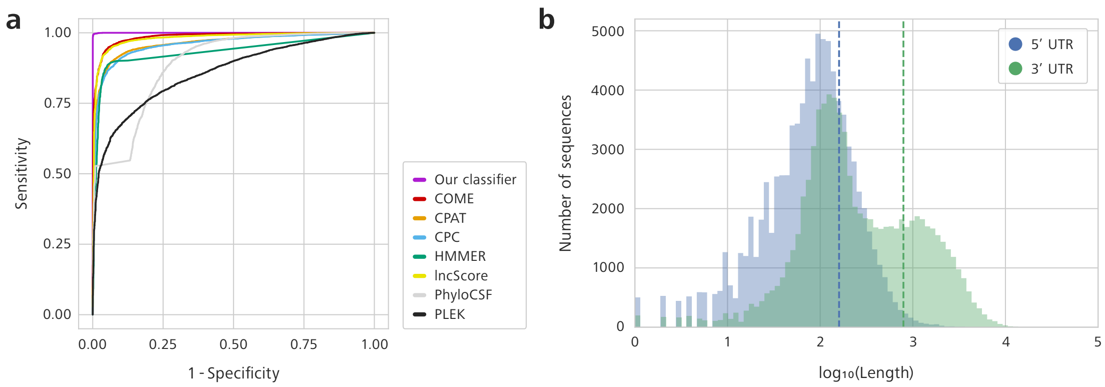

Figure S2. (a) ROC curve of our lncRNA classifier (purple) in comparison with other currently available classification models. (b) Histograms of 5' (blue) and 3' (green) UTR length (in number of base pairs) distribution in the mouse transcriptome. The dashed vertical lines mark the upper quartile values of each distribution.

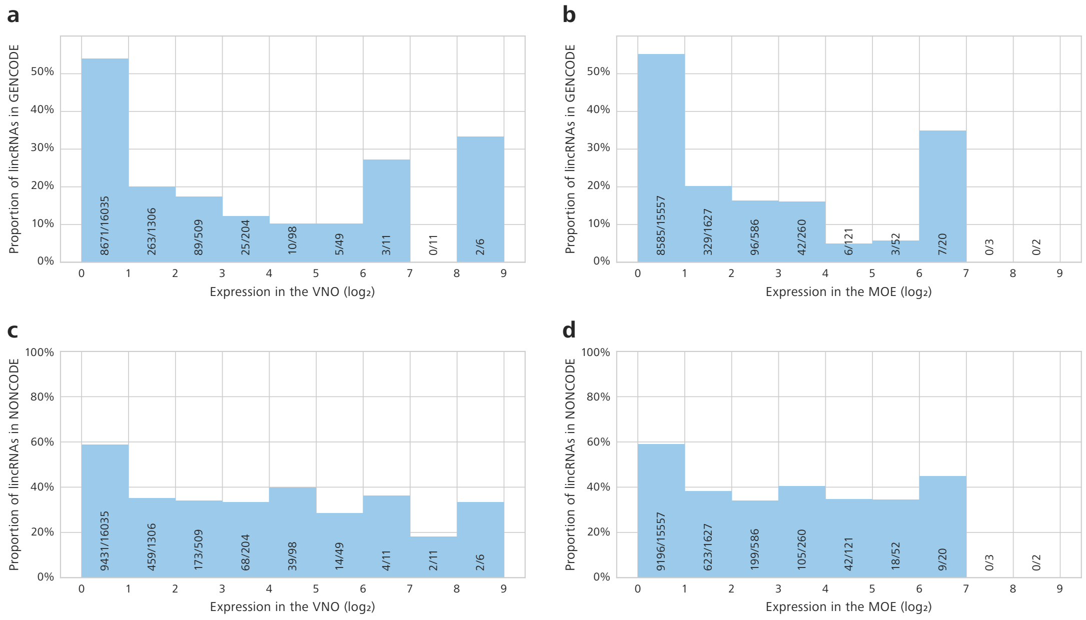

Figure S3. Proportion of annotated lncRNAs grouped by their expression levels in the olfactory organs. The bars height (y-axis) represents the proportion of identified lncRNAs that were previously annotated in (a, b) GENCODE M9 and (c, d) NONCODE v5. Raw transcript numbers are displayed over the bars. Expression level intervals (in TPM) in the (a, c) VNO and (b, d) MOE are represented in the x-axis. The majority of highly expressed lncRNAs in the VNO and MOE are still unannotated, further evidencing that the transcriptomes of these tissues are still mostly unknown.

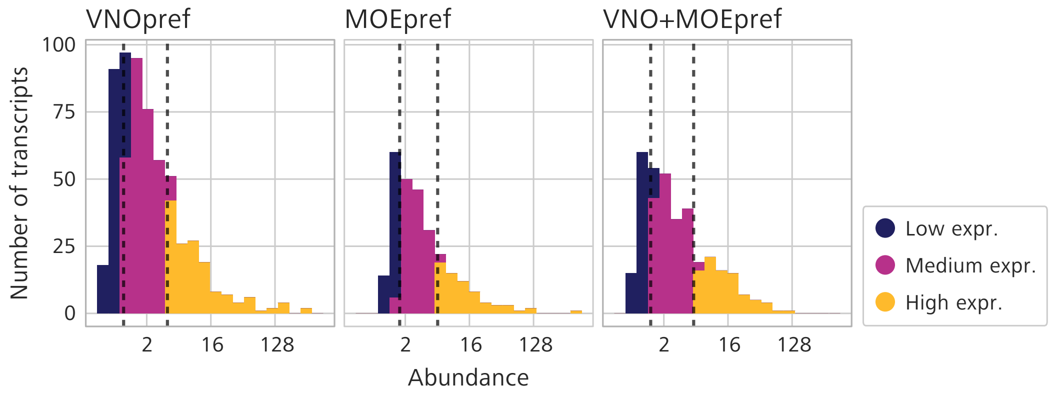

Figure S4. lncRNA expression abundance groups. Histograms represent the distribution of the expression abundances (x-axis) for the entire olfactory lncRNA dataset in the VNO_{pref}, MOE_{pref} and $VNO{+}MOE_{pref}$ preferential expression groups. Vertical dashed lines represent the quartiles of expression for each distribution. Transcripts below the lower quartile were considered "low expression" lncRNAs (dark blue transcripts); those lying in the interquartile range were considered "medium expression" lncRNAs (magenta); and transcripts above the upper quartile were placed in the "high expression" group (yellow). The same color scheme is shown for *in situ* hybridization for selected genes in Fig. 3.

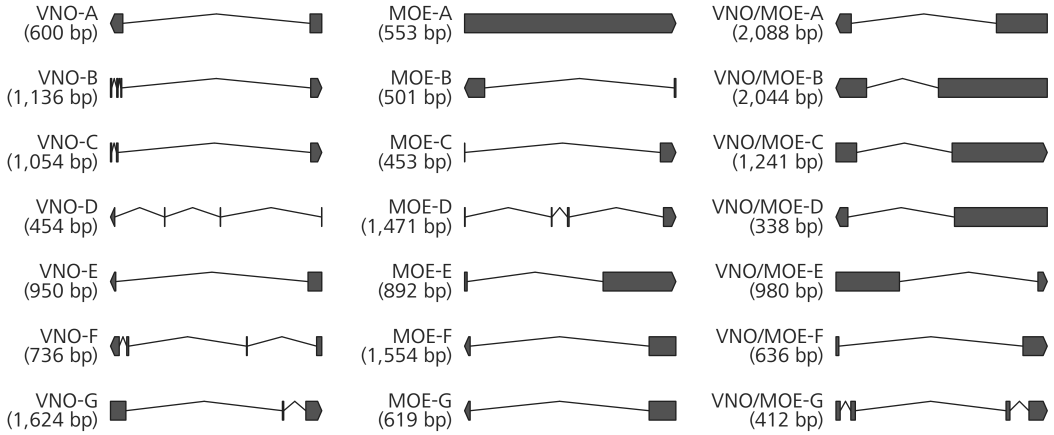

Figure S5. Representation of lncRNA gene structure models. Gene models for lncRNAs selected for further validation in the VNO_{pref} (right), MOE_{pref} (center) and $VNO+MOE_{pref}$ (right) groups. For each lncRNA transcript, the size shown in parenthesis refers to the combined size of spliced exons. Exons are shown as gray boxes and introns as cuspid lines. The orientation of each transcript in the mouse genome is depicted with the arrow: rightward arrow indicates the lncRNA is on the + (plus) strand, while the leftward arrow refers to a transcript produced from the – (minus) strand, according to the GRCm38 mouse genome. Sizes are not comparable across transcripts

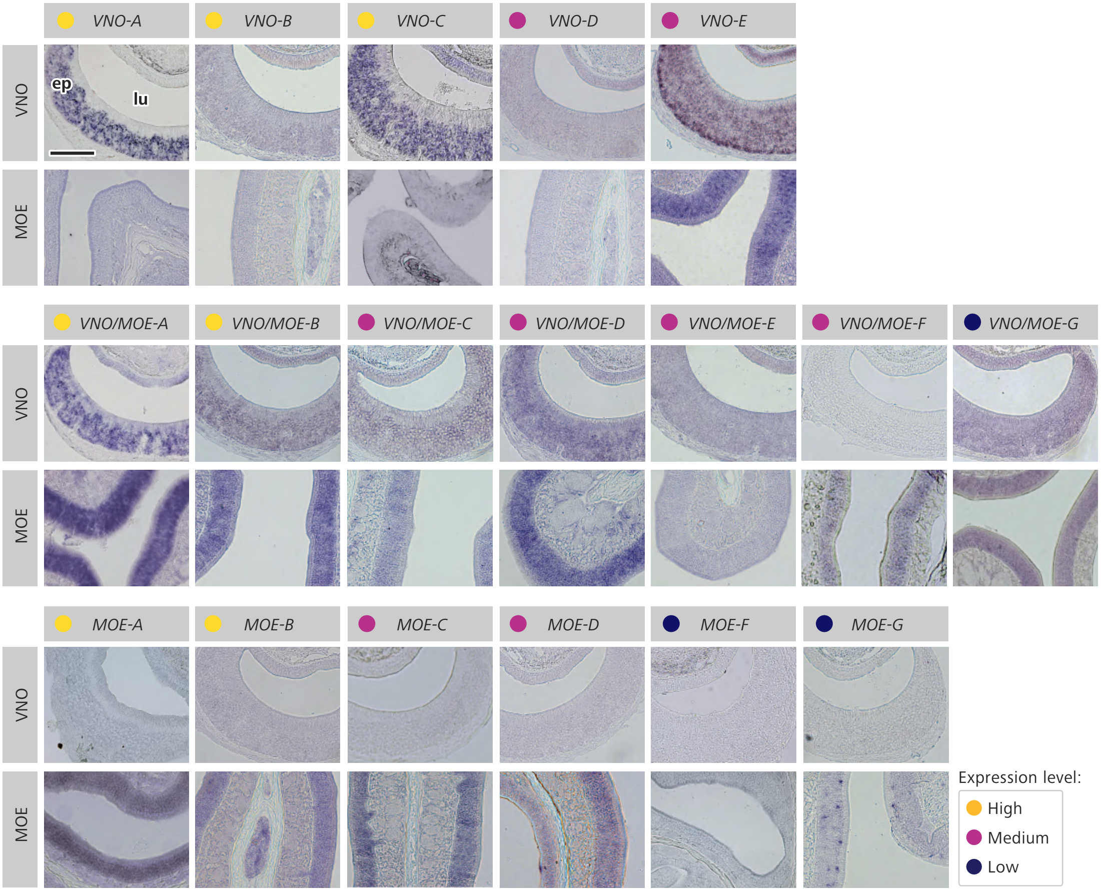

Figure S6. High magnification microscopy images of olfactory tissue sections subjected to chromogenic in situ hybridization staining (purple) with riboprobes for selected preferentially-expressed lncRNAs. Expression level groups 'high', 'medium' and 'low' were determined according to expression quartiles (Supplementary Fig. S4) and are indicated by yellow, magenta, and blue circles, respectively. lu, VNO lumen; ep, MOE or VNO sensory epithelia. Scale bar = 100 µm.

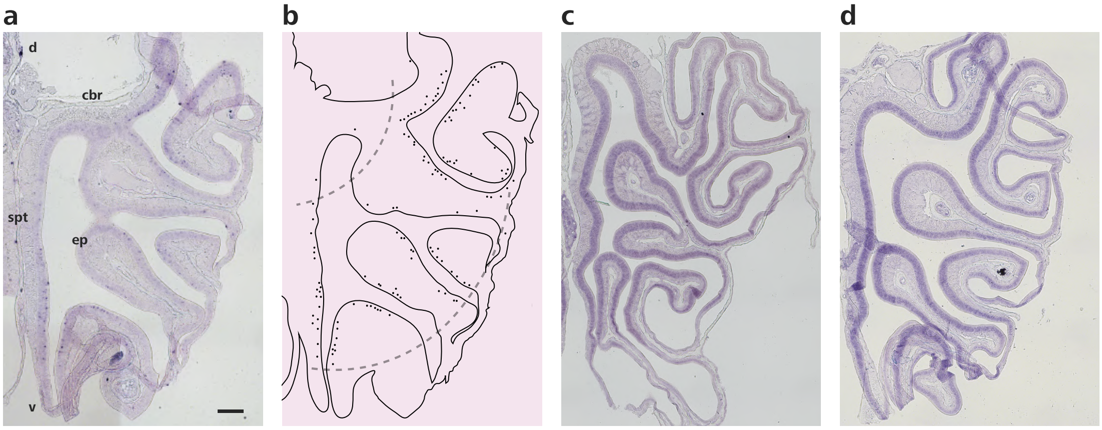

Figure S7. Zonal distribution of lncRNA *MOE-G* punctate pattern of expression. (a) Low magnification composite image showing *in situ* hybridization signal (purple staining) for lncRNA *MOE-G*. Note the expression in a subset of cells in the MOE neuroepithelium (punctate staining). The MOE's dorsal side faces up. Only the right side of the MOE is shown in this image (the nasal septum lies to the left). (b) Schematic representation of the distribution of *MOE-G*-positive cells (dots) in the MOE. Stained cells are clearly non-randomly distributed across the MOE turbinates, being more frequent in intermediate zones of the MOE (between the dashed lines). Note the almost absent staining in the most dorsal and ventral MOE zones. (c, d) Low magnification images of *VNO/MOE-B* (c) and *VNO/MOE-D* (d), showing expression in the MOE sensory epithelium across all turbinates, without discernible zonation. cbr, cribriform plate; spt, nasal septum; ep, MOE epithelium; d, dorsal; v, ventral. Bar is 100 µm.

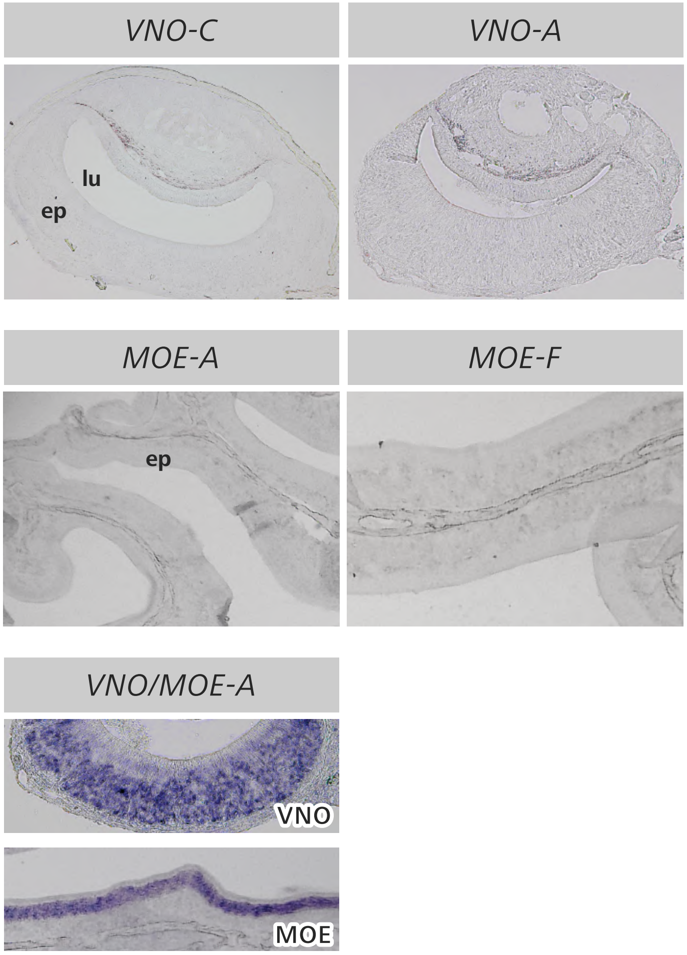

Figure S8. *In situ* hybridization with control sense cRNA probes. Representative microscope images of MOE tissue sections subjected to chromogenic *in situ* hybridization staining with control sense riboprobes for selected preferentially-expressed lncRNAs. Note that for *VNO/MOE-A* the sense probe also detected transcripts in the VNO and MOE. ep, sensory epithelium, lu, VNO lumen. Size bar indicates 100 µm.

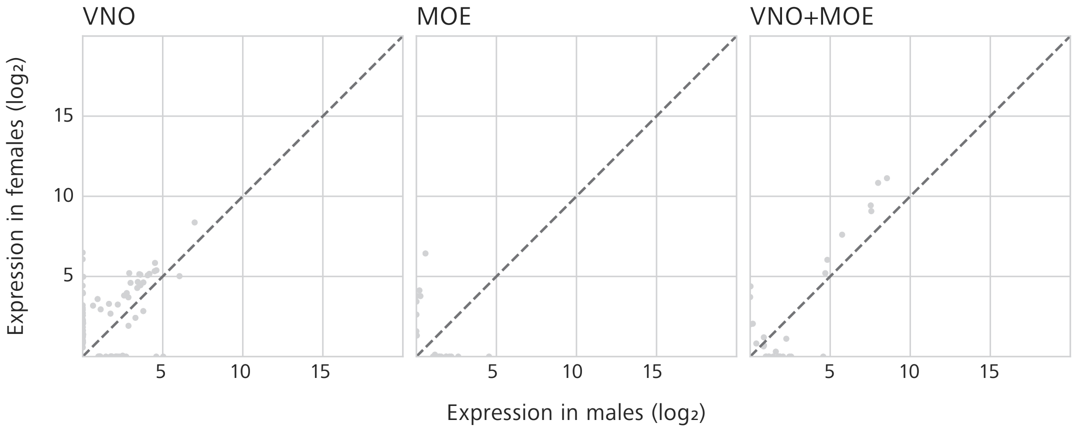

Figure S9. Mean expression levels (in TPM) of VNO_{pref}, MOE_{pref}, and VNO+MOE_{pref} transcripts in males (x-axis) and females (y-axis). Each gray dot represents a transcript that is differentially expressed between the genders. No lincRNA belonging to either one of these groups was differentially expressed.

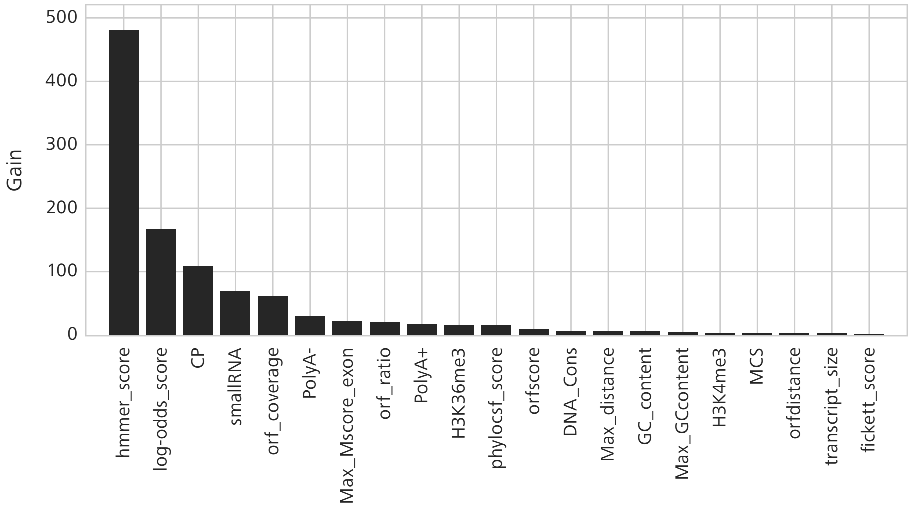

Figure S10. Importance of the features used by our lncRNA classifier. hmmer_score, which is not used by any other machine learning lncRNA classifier, was shown to be the most important feature for lncRNA identification. Feature importance was quantified as the average gain in accuracy brought by the feature across all decision tree splits it is used in.

Table S1. RNA-seq libraries used for transcriptome assembling and identification of olfactory-preferential lincRNAs.

SRA id	Tissue/organ	Reference
SRR636961 to SRR636963	Brain	[1]
SRR306757 to SRR306761	Brain	[2]
SRR306763 and SRR306764	Cerebellum	[2]
SRR531315 to SRR531317	Cerebral cortex	[3]
SRR306766 and SRR306767	Heart	[2]
SRR636916 to SRR636918	Kidney	[1]
SRR306769 and SRR306770	Kidney	[2]
SRR636868 to SRR636870	Liver	[1]
SRR306772 and SRR306773	Liver	[2]
ERR225908 to ERR225913	MOE (adults)	[4]
ERR323395 to ERR323397,<br>ERR323401 to ERR323403	MOE (newborns)	[5]
SRR2961020 to SRR2961112	MOE (single-cell)	[6]
ERR036348 to ERR036353	VNO (adults)	[4]
ERR323398 to ERR323400,<br>ERR323404 to ERR323406	VNO (newborns)	[5]

Table S2. Description of the features used by our lncRNA identification model and the software responsible for computing each of them.

**Feature**	**Description**	**Software**
DNA_Cons	Evolutionary conservation of transcript sequence, quantified by the phastCons algorithm [7].	COME
PolyA-	Expression in poly-A depleted RNA-seq libraries.	COME
PolyA+	Expression in poly-A enriched RNA-seq libraries.	COME
smallRNA	Expression in small RNA sequencing libraries.	COME
GC_content	Proportion of G and C nucleotides in the transcript sequence.	COME
H3K36me3	Proximity to H3K36me3 histone proteins.	COME
H3K4me3	Proximity to H3K4me3 histone proteins.	COME
transcript_size	Number of nucleotides in the transcript.	CPAT
fickett_score	Score based on the differences in preferential position of each nucleotide between coding and non-coding transcripts [8].	CPAT, lncScore
log-odds_score	ORF quality assesment.	CPC
orf_coverage	ORF quality assesment.	CPC
hmmer_score	Full sequence log-odds score computed by hmmsearch.	HMMER
orf_ratio	Ratio between the ORF and the transcript lengths.	lncScore
orfscore	Score based on hexamer frequencies in the ORF.	lncScore

orfdistance	Average distance between the orfscore of the three reading frames.	lncScore
Max_Mscore_exon	Higher orfscore value among the exons.	lncScore
Max_distance	Higher orfdistance value among the exons.	lncScore
Max_GCcontent	Higher GC content value among the exons.	lncScore
MCS	orfscore of the maximum coding subsequence.	lncScore
CP	Ratio between the higher MCS among the three reading frames and their summation.	lncScore
phylocsf_score	Coding potential score based on codon evolutionary patterns.	PhyloCSF

Table S3. Strand, median abundance (TPM) and tissue-specificity (SPM) of marker genes in the MOE and VNO of adult mice.

Gene	Strand	MOE		VNO	
		Abundance	SPM	Abundance	SPM
Omp	−	2483.85	0.65	532.73	0.53
Cnga2	+	321.10	0.95	6.06	0.32
Trpc2	+	1.25	0.18	84.41	0.96

Table S4. Pairs of primers used to amplify selected lncRNAs from mouse cDNA. All sequences are in the 5' to 3' orientation.

**Transcript**	**Forward**	**Reverse**
VNO-A	CCACTGATTGCAGCTGATG	TGAATAAAATTGTGGTTTCGGATGAC
VNO-B	TGTACTCTGAGACGTGAGACGGTC	GCTCAGAAAGCAAACATCCAAGGAG
VNO-C	GAGCCGAGCTTCTCCTC	GAAGGGTGCTGGAATCTC
VNO-D	ATGGGATTCTTTCCAGCTTCACGTC	ACGCCACAATCCAGCTATCTTCTG
VNO-E	TTCTCACCACCGCTTATAATGGCTC	CTTCCAAATACTCTGGCACACACAC
VNO-F	CGCCCATCTGAGATGAAATTGCAG	CTGTTGCAGTCAGCTCTATCAGTG
VNO-G	ACAACACTTGTCGCTGTTGGTACTG	AGGGAAACAACGCCAGACTCCAC
MOE-A	GTAGAGAGTCCTTAGTAACCTGGAACTC	GAAAGTGGCCAGGAAAGACTG
MOE-B	TGAAAGAGACTGGTGTTTGGTTACGTG	AGCCTCTTCAGAGAAGGCACTG
MOE-C	GATTCTCACGGAGGAAGGAGCAC	TCACACTCTGTCTGTGGATGGGAG
MOE-D	CAGAGACGTGCAGTAACTCTGGAG	AAGCAAAGAGCCCAACAGGCTC
MOE-E	AAAGTTAAGGAAAGGTAGAAGAGGTGAG	AGCACATCCAGTATAGTTGGGTC
MOE-F	TCTCCTTCGCAACCAGATCG	AATCATCTTGACTTCAGAACCACTG
MOE-G	GCCAGTCTTATCGTGAGGAGCTG	AGTCCTGTGGATACAGCTATGCAG
VNO/MOE-A	CTACGTCTTCCAACGTGAACCAC	GTGCTAGGGAACAATGAAGCTG
VNO/MOE-B	TGGGTTGTGGAGAAACAAATAAGGCAG	CAGGAGAAACAGTGGGCATTAGGAC
VNO/MOE-C	AGGCGATGAGAAAGTGTACCTAAGAG	CACCAACAGATGCTGTGCTGTCTG
VNO/MOE-D	CGTTGATGGTGTACCCTAACTCCAG	GGGTATAATTTGGGCGGCAGCTG
VNO/MOE-E	TCATCACACGTCACTGAATAGCGTC	GAGACATGCACACCAATCCACTCAG
VNO/MOE-F	TTCTCTTCGCTTGTCTGCCGAG	CAAGGGATCCAAGCAGGAGACTG
VNO/MOE-G	GTTTTGCAGGGCTGTGTTTCATCTC	GGTGGAGCTGATGTGATAAGCACTC

Table S5. Number of differentially expressed transcripts (male vs. female) detected in each of the three comparisons.

	**Total**	**Preferentially expressed lncRNAs**
VNO+MOE	29	0
MOE	21	0
VNO	95	0

Supplementary methods

RNA-Seq library selection and quality control

Raw sequencing data was obtained from the SRA (Sequence Read Archive) database, maintained by the NCBI (National Center for Biotechnology Information). We carefully selected libraries from a variety of non-diseased tissues and organs from C57BL/6 mice. All libraries had at least two technical replicates. We collected data from five different studies [1–5], totaling 49 samples, of which 43 are from adult mice (brain, cerebellum, cerebral cortex, heart, kidney, liver, MOE and VNO) and 6 are from newborn animals (MOE and VNO). SRA identifiers, the tissues from which the libraries were prepared, and the number of samples in each library are reported in Table S1.

The MOE and VNO RNA-seq libraries from newborn mice were sequenced with technical replicates. As a PCA analysis showed that the sequencing lane had no observable effect over the abundance of the transcripts, the FASTQ files of the technical replicates of each biological sample were concatenated.

It is important to note that none of the libraries are stranded, that is, the information of the strand from which the transcripts are transcribed is lost. This choice was done because the most important libraries in our study (VNO and MOE libraries), with which all others would be compared, are not directional.

We also used single cell RNA-seq libraries obtained from 93 dissociated olfactory neurons from the MOE sensory epithelium [6] (Table S1).

Transcriptome assembly

As we were interested in identifying novel lincRNAs, we decided not to use publicly available mouse genome annotation from GENCODE [9]. Instead, the transcriptome was assembled by mapping RNA-Seq reads from the adult mouse samples to the GRCm38 primary assembly masked version, downloaded from Ensembl [10].

Splicing-aware read mapping was performed using STAR (version 2.5.1b) [11], using the parameter 'sjdbGTFfile', which gathers exon-intron splice junction from the annotation of the mouse genome (GENCODE's comprehensive gene annotation release M9) to increase mapping accuracy. A 2-pass mapping step was performed using the 'sjdbFileChrStartEnd' parameter to gather splice junctions detected during the first mapping step. Transcriptome assembly for each sample was performed using Cufflinks (version 2.2.1) [12], and Cuffmerge was used to merge together the resulting assemblies, using the 'ref-gtf' parameter to include GENCODE's comprehensive gene annotation (release M9), which improves the final assembly and allowed

automated gene annotation.

The stability of the Cufflinks' assembly was checked by comparison with an alternate assembly built with StringTie (version 1.3.5) [13]. The comparison between the assemblies was performed using GffCompare (version 0.11.0). Loci that were assigned to the "=", "c", or "k" classes were considered equivalent between the two assemblies.

Candidate lincRNA identification in the assembled transcriptome

For the machine learning training phase, we built a dataset set by selecting transcripts annotated as 'lincRNA' or 'protein_coding' in the field 'gene_type' in the GTF file from the GENCODE comprehensive genome annotation (release M11). The dataset was then divided into a test and training set (with 20% and 80% of the total transcripts, respectively) containing the same proportion of coding and non-coding transcripts.

The features used for training the classification model were obtained from the following software: COME (version 1), CPAT (version 1.2.4), CPC (version 0.9 r2), HMMER (version 3.1 b2), lncScore (version 1.0.2) and PhyloCSF. After the selection of informative variables, 21 features were chosen for model training (Supplementary Table S2). The training set, containing 10,956 lincRNAs and 46,743 protein coding transcripts, was used to train a tree ensemble model using the XGBoost algorithm (version 0.6 of the xgboost Python package). To evaluate the classification performance, the transcripts in the test set (containing 2,739 lincRNAs and 11,686 protein coding transcripts) were classified using the trained model and the following metrics were computed: accuracy, sensitivity, specificity, precision, area under the precision-recall curve and area under the ROC curve.

Finally, this model was used to classify the transcripts assembled by Cufflinks with length $\geq$ 200 nt into lncRNA or protein-coding transcripts. Transcripts that were not assigned to a specific strand during assembly were tested for both strands. Only the transcripts that had both strands classified as non-coding were considered lncRNAs. More details concerning feature computation, feature selection, training and evaluation of the classification model can be found in the Supplementary Computational Notebook.

Because we were interested in intergenic lncRNAs (lincRNAs), we further filtered the candidate dataset by choosing those transcripts that showed no genomic overlap with other loci, including genes for tRNAs, miRNAs, snoRNAs, pseudogenes, intronic lncRNAs, reverse-strand lncRNAs, and lncRNAs that share the same strand with other coding genes, such as non-coding splicing variants, as performed elsewhere [14]. To this end, we obtained a GTF file of the GENCODE comprehensive genome annotation (release M11) and selected transcripts with the following classifications in the 'gene_type' field, to generate a new GTF file containing the unwanted loci: 'pro-

tein_coding', 'nonsense_mediated_decay', 'non_stop_decay', 'IG_*_gene', 'TR_*_gene', 'polymorphic_pseudogene', 'miRNA', 'misc_RNA', 'Mt_rRNA', 'Mt_tRNA', 'ribozyme', 'rRNA', 'scaRNA', 'scRNA', 'snoRNA', 'snRNA', 'sRNA', 'IG_C_pseudogene', 'IG_D_pseudogene', 'IG_pseudogene', 'IG_V_pseudogene', 'processed_pseudogene', 'transcribed_processed_pseudogene', 'transcribed_ unitary_pseudogene', 'transcribed_ unprocessed_pseudogene', 'translated_ processed_pseudogene', 'TR_J_pseudogene', 'TR_V_pseudogene', 'unitary_pseudogene', 'unprocessed_pseudogene'. Then, transcripts in the transcriptome assembled by Cufflinks that displayed genomic overlap with these unwanted gene loci were removed via the 'intersect' function of bedtools (version 2.26.0) [15]. GENCODE also provides GTF files with pseudogenes (consensus pseudogenes predicted by the Yale and UCSC pipelines) and tRNAs (Predicted tRNA genes). These files were also used in two further steps of removal of genome overlapping transcripts.

Finally, it is important to keep in mind that transcription reconstruction software typically return fragments of untranslated regions of mRNAs (UTR) as independent transcripts [14], which can be misclassified as non-coding. As not all coding transcripts annotated in GENCODE have annotated UTR regions, it is possible that fragments of UTRs escaped the first filtering step. Therefore, an additional filter was performed to avoid false positives. For this step, it was necessary to obtain genomic intervals for 5' and 3' UTRs that are representative of the mouse genome. To determine representative lengths of the 5' and 3' UTR, we first obtained the sequences of all annotated UTRs in the Ensembl database (release 87) using the biomaRt software (version 2.30) [16]. Next, the length distributions of the sequences obtained were inspected (Fig. 2). As many recorded UTRs are extremely short, probably due to annotation errors, the mean or median would not be representative of UTR lengths. Therefore, we decided to use the upper quartile of the distributions – 160 bp (5' UTR) and 792 bp (3' UTR) – to generate a GTF file containing intervals corresponding to the annotated coding regions plus the representative length values for the UTRs (artificial coding transcripts). We excluded one-exon transcripts that showed any overlap with the artificial coding transcripts. For transcripts with multiple exons, we excluded only transcripts whose overlap with the artificial coding transcripts comprised more than 25% of its length (parameter 'f').

Quantification of transcript expression

Kallisto index was generated from the Cuffmerge assembled transcriptome. The abundances of transcripts were estimated using the 'bias' parameter to correct the quantification for sequence bias [17], and the parameter 'bootstrap-samples' to generate 100 bootstrap samples during the expectation-maximization step. For samples with unpaired reads (single-end), the parameters 'fragment-length' and 'sd' were set to 200 bp and 80 bp, respectively [18]. Between-sample abundance normalization was applied to the raw abundance data (TPM values) using library size factors as computed by sleuth (version 0.30.0) [19]. Gene-level abundance was obtained

summing up the normalized abundances of all isoforms.

RNA sequencing using the SOLiD platform

Ribosomal RNA was depleted from total RNA prepared from olfactory epithelia from newborn (P3) and 4 weeks old mice using the RiboMinus Eukaryote Kit for RNA-seq (Invitrogen). RNA-Seq libraries were prepared using SOLiD Total RNA-Seq Kit, according to the manufacturers' recommendations, and were sequenced on the SOLiD sequencing platform (Life technologies, Carlsbad, CA). The obtained strand-specific reads were mapped to a color-space converted GRCm38 primary assembly masked version using BWA-backtrack (version 0.5.9) [20] and transcripts were assembled using Cufflinks (version 2.2.1) and Cuffmerge. Then, Cuffcompare was used to compare the SOLiD assembly to the one generated using Illumina reads (described in "Transcriptome assembly") and strand information was assigned to strandless transcripts that matched SOLiD assemblies.

Identification of transcripts preferentially expressed in the olfactory organs

To quantify how specific was the expression of a transcript in a given tissue we used the tspex [21] package to compute the SPM (specificity measure) metric. The SPM value of a transcript 'x' in a tissue 'i' corresponds to the similarity of the cosine between the vector with the of expression of 'x' in all tissues and a vector with exclusive expression of 'x' in tissue 'i'. The choice of SPM is due to the fact that this measure shows the specific expression separately for each tissue, a property of great importance in our work, since we wanted to select not only transcripts that are preferentially expressed in the MOE (MOE_{pref}) or VNO (VNO_{pref}), but also those transcribed preferentially in both olfactory tissues relative to other tissues analyzed ($MOE+VNO_{pref}$). Transcripts whose values of normalized abundance in all libraries were smaller than 1 were excluded from the analysis. SPM was calculated using the $\log_2$(normalized abundance + 1) values at both transcript and gene levels. The median expression of each transcript/gene among the samples of a given library was used as a representative value of the expression in that tissue or organ.

Identification of transcripts differentially expressed in olfactory organ libraries

Differential expression tests were performed to identify differentially expressed transcripts between two biological conditions (male vs. female and adult vs. newborn). For this, the kallisto

expression quantification incorporating bootstrap data was analyzed with sleuth (version 0.28.1), using models with covariates indicating the tissue (MOE or VNO), sex (male or female) and developmental stage (adult or newborn). Likelihood ratio tests were performed at the transcript level and differentially expressed transcripts were selected at a 5% false-discovery rate threshold.

Given the relevance of the modular architecture of lincRNAs in determining their function, and the role of alternative splicing in the generation of isoforms with different combinations of modules, it was decided to run the differential expression tests at the transcript level. The resulting loss of sensitivity was not detrimental, since only a few transcripts would be chosen for experimental validation.

Analysis of MOE single-cell RNA-Seq libraries

The analysis of MOE scRNA-Seq data was done with Monocle (version 2.10.0) [22]. Since each sample from the scRNA-Seq library corresponds to a single cell and the MOE cells are heterogeneous, as they represent different stages of the neurogenesis process, cells can be ordered along a path of cellular differentiation. Given that the gene expression profiles change continuously throughout neurogenesis, Monocle uses abundance data to order the expression patterns along a pseudotime, a quantitative measure of progress along a continuous biological process.

Initially, raw abundance data (as TPM) of the 93 scRNA-Seq samples was converted to absolute abundance (estimated number of RNA molecules per cell) using the built-in Census algorithm ('relative2abs' function) [23]. Next, the transcripts whose expression data would be used to reconstruct the differentiation path were selected in an unsupervised manner by selecting transcripts with empirical dispersion greater than the fitted dispersion model and absolute abundance greater than 0.5. Using DDRTree, the absolute abundance matrix of the selected transcripts was reduced to a two-dimensional space ('reduceDimension' function), in which the path of the pseudotime underlying the expression data was laid [24]. Then, the algorithm assigned the position of each cell in that path, that is, the pseudotime associated with that sample ('orderCells' function). As the reconstruction of the route was done in an unsupervised way, we defined which end of the pseudotime corresponds to the beginning (precursor cells) and the end (mature neurons) of neurogenesis, based on the expression of *Ascl1* (precursor cell marker) and *Cnga2* (MOE mature sensory neuron marker).

Finally, we performed likelihood ratio tests with the 'differentialGeneTest' function to identify transcripts that are differentially expressed between OMP-positive and OMP-negative cells. We labeled as OMP-positive the cells in which the absolute abundance of the *Omp* gene was higher than 100. Transcripts were selected at a 5% false-discovery rate threshold and the ones with higher average expression in the OMP-positive cells were chosen for further analysis. Smooth spline curves representing transcript expression dynamics along pseudotime was obtained

for the selected transcripts using the 'genSmoothCurves' function.

cDNA synthesis and RT-PCR

We selected candidate lincRNAs for experimental validation and further investigation based on estimated abundance levels. VNO-specific, MOE-specific and olfactory-specific transcripts were chosen that have high TPM values and are not positioned close to other genes, avoiding the inadvertent selection of coding gene fragments. Given that lincRNAs commonly act as cis-regulatory elements, preference was given to transcripts located near ORs or VRs receptors. As many lincRNA loci transcribe multiple transcripts, we chose which isoform best represents the mapping pattern of reads from 'MOE' and 'VNO' libraries. To this end, we used Integrative Genomics Viewer software, version 2.3, to perform a careful inspection of transcript assemblies, considering coverage of reads in all libraries, and only one transcript was chosen per lincRNA locus.

Synthesis of cDNA was performed of RNA extracted from VNO, MOE, liver and mouse brain, using the ImProm-II™ Reverse Transcription System (Promega Corporation), following the manufacturer's recommendations. One microgram of RNA sample was added to a 0.2 mL together with 500 ng of oligo-dT20. The tubes were kept at 70°C for 5 min and then transferred to ice for another 5 min, followed by addition of 4 µL of 5x reaction buffer, 1.5 µL 25 mM MgCl2, 1 µl dNTP Mix (0.5 mM), 0.5 µL of ribonuclease inhibitor RNasin (Promega Corporation), 1 µl of ImProm-II™ reverse transcriptase and 7 µL of nuclease-free water. Samples were then maintained at 25°C for 5 min, at 42°C for 2 h, at 70°C for 15 min, and then stored at -20°C. PCR primers were designed in exons near the ends of the transcripts, to capture whatever splicing arrangements between the first and the last exon, which could be identified through Sanger sequencing of the amplified fragments. To amplify the selected lincRNAs by PCR, we added to a 0.2 tube 5 µl GoTaq® enzyme buffer, 1.5 µL 25 mM MgCl2, 0.5 µL of dNTP Mix (10 mM), 1 µl of lincRNA forward primer (5 µM), 1 µL of the corresponding reverse primer (5 µM), 0.25 µL GoTaq® enzyme, and 2 µl cDNA sample. Reactions were subjected to the following amplification cycling parameters: 94 oC for 5 min, followed by 35 cycles of 1 min at 94°C, 1 min at the adequate annealing temperature and 2 min at 72°C.

In situ hybridization

The dissected VNOs were immersed in 4% paraformaldehyde fixative solution (in 1x PBS) at 4°C overnight. Then, the samples were transferred to a demineralizing solution (0.45M EDTA, 1x PBS) for a period of 4 h, followed by incubation in cryoprotectant solution (30% sucrose) for another two hours. VNOs were placed in O.C.T. Tissue-Tek® (Sakura Finetek) and sectioned on a Leica CM1850 cryostat to produce 12 µm sections. MOE samples were processed the same

way, except that demineralization proceeded for 10 h and cryoprotection was done for 24 h.

The VNO and MOE sections were air-dried for 10 min, fixed in 4% paraformaldehyde for 20 min, permeabilized with 0.1 M HCl for 10 min, and acetylated with 0.1 M triethanolamine (pH 8.0) containing acetic anhydride for 10 min. Slides were washed twice in 1×PBS between incubations. Hybridization was done with DIG-labelled probes (1.5 µg/mL) at 58°C in hyb. solution (50% formamide, 10% dextran sulfate, 600 mM NaCl, 200 µg/mL yeast tRNA, 0.25% SDS, 10 mM Tris-HCl pH 8.0, 1×Denhardt's solution, 1 mM EDTA pH 8.0) for 16 h. Slides were washed in a series of baths of increasing stringency (2× SSC, 0.2×SSC and 0.1×SSC) at 60°C, 20 min each. Slides were then incubated in 0.1% Tween-20 for 10 min, washed in TN buffer (100 mM Tris-HCl pH 7.5, 150 mM NaCl) for 10 min at room temperature, and subjected to immunostaining for DIG. This was performed by blocking in TNB buffer (100 mM Tris-HCl pH 7.5, 150 mM NaCl, 0.05% Perkin Elmer blocking reagent), incubation at 4°C for 16 h with rabbit anti-DIG (Roche) primary antibody diluted 1:600 in TNB buffer, and development using BCIP and NBT.

For some double *in situ* experiments, we combined chromogenic *in situ* detection for lncRNA with fluorescent *in situ* detection for *Omp* or *Ki67*, because chromogenic development is more sensitive, ensuring that the full complement of lncRNA-positive cells was labeled. Complementary RNA probes were synthesized with rNTPs labeled with either DNP or DIG (Roche), from fragments amplified to include suitable SP6 or T7 RNA polymerase promoters. Sixteen micrometer sections were air-dried for 10 minutes, fixed in 4% paraformaldehyde for 20 min, and treated with 0.1 M HCl for 10 min, and with 250 mL of 0.1M triethanolamine (pH 8.0) containing 1mL of acetic anhydride for 10 min. Hybridization was performed with DNP (1 μg/mL) or DIG (600 ng/mL) labeled cRNA probes at 60°C in 50% formamide, 10% dextran sulfate, 600mM NaCl, 200µg/ml yeast tRNA, 0.25% SDS, 10mM Tris-HCl pH 8.0, 1X Denhardt's solution, 1mM EDTA pH 8.0. Washes were done in 2x SSC, 0.2x SSC and 0.1x SSC at 60°C. After blocking in TNB buffer (100 mM Tris-HCl pH 7.5, 150 mM NaCl, 0.05% BSA), slides were incubated at 4°C with rabbit anti-DNP primary antibody diluted 1:600 in TNB buffer. Signal development with tyramide was followed by tyramide-biotin, streptavidin-HRP, and tyramide-Alexa 546 incubations. Slides were then blocked again in TNB, followed by incubation with anti-DIG-POD and signal development was performed using BCIP and NBT in the presence of 5% PVA. Samples were stained with To-Pro 3 (Invitrogen) and mounted [25].

For double fluorescent *in situ* hibridization, we used DIG and DNP-labeled cRNA probes for *VNO-E* and *Ki67*, respectively. DNP signal development was performed as described above. For DIG-labeled probes, we used tyramide signal amplification as previously described [25]. Images were collected on a Leica TCS SP5 II confocal microscope using optical sections of 0.896 µm.

References

1. Fushan, A. A. *et al.* Gene expression defines natural changes in mammalian lifespan. *Aging cell* **14,** 352–365 (2015).
2. Brawand, D. *et al.* The evolution of gene expression levels in mammalian organs. *Nature* **478,** 343 (2011).
3. Dillman, A. A. *et al.* mRNA expression, splicing and editing in the embryonic and adult mouse cerebral cortex. *Nature neuroscience* **16,** 499 (2013).
4. Ibarra-Soria, X., Levitin, M. O., Saraiva, L. R. & Logan, D. W. The olfactory transcriptomes of mice. *PLoS genetics* **10,** e1004593 (2014).
5. Ibarra-Soria, X. *et al.* Variation in olfactory neuron repertoires is genetically controlled and environmentally modulated. *Elife* **6,** e21476 (2017).
6. Hanchate, N. K. *et al.* Single-cell transcriptomics reveals receptor transformations during olfactory neurogenesis. *Science* **350,** 1251–1255 (2015).
7. Siepel, A. *et al.* Evolutionarily conserved elements in vertebrate, insect, worm, and yeast genomes. *Genome research* **15,** 1034–1050 (2005).
8. Fickett, J. W. Recognition of protein coding regions in DNA sequences. *Nucleic acids research* **10,** 5303–5318 (1982).
9. Harrow, J. *et al.* GENCODE: the reference human genome annotation for The ENCODE Project. *Genome research* **22,** 1760–1774 (2012).
10. Zerbino, D. R. *et al.* Ensembl 2018. *Nucleic acids research* **46,** D754–D761 (2017).
11. Dobin, A. *et al.* STAR: ultrafast universal RNA-seq aligner. *Bioinformatics* **29,** 15–21 (2013).
12. Trapnell, C. *et al.* Transcript assembly and quantification by RNA-Seq reveals unannotated transcripts and isoform switching during cell differentiation. *Nature biotechnology* **28,** 511 (2010).
13. Pertea, M. *et al.* StringTie enables improved reconstruction of a transcriptome from RNA-seq reads. *Nature biotechnology* **33,** 290 (2015).

14. Chen, J. *et al.* Evolutionary analysis across mammals reveals distinct classes of long non-coding RNAs. *Genome biology* **17,** 19 (2016).

15. Quinlan, A. R. & Hall, I. M. BEDTools: a flexible suite of utilities for comparing genomic features. *Bioinformatics* **26,** 841–842 (2010).

16. Durinck, S., Spellman, P. T., Birney, E. & Huber, W. Mapping identifiers for the integration of genomic datasets with the R/Bioconductor package biomaRt. *Nature protocols* **4,** 1184 (2009).

17. Roberts, A., Trapnell, C., Donaghey, J., Rinn, J. L. & Pachter, L. Improving RNA-Seq expression estimates by correcting for fragment bias. *Genome biology* **12,** R22 (2011).

18. Trapnell, C. *et al.* Differential gene and transcript expression analysis of RNA-seq experiments with TopHat and Cufflinks. *Nature protocols* **7,** 562 (2012).

19. Pimentel, H., Bray, N. L., Puente, S., Melsted, P. & Pachter, L. Differential analysis of RNA-seq incorporating quantification uncertainty. *Nature methods* **14,** 687 (2017).

20. Li, H. & Durbin, R. Fast and accurate short read alignment with Burrows–Wheeler transform. *bioinformatics* **25,** 1754–1760 (2009).

21. Camargo, A. P. *tspex: tspex: tissue-specificity calculator.* `https://github.com/apcamargo/tspex`.

22. Qiu, X. *et al.* Reversed graph embedding resolves complex single-cell trajectories. *Nature methods* **14,** 979 (2017).

23. Qiu, X. *et al.* Single-cell mRNA quantification and differential analysis with Census. *Nature methods* **14,** 309 (2017).

24. Mao, Q., Wang, L., Goodison, S. & Sun, Y. *Dimensionality reduction via graph structure learning* in *Proceedings of the 21th ACM SIGKDD International Conference on Knowledge Discovery and Data Mining* (2015), 765–774.

25. Nakahara, T. S. *et al.* Detection of pup odors by non-canonical adult vomeronasal neurons expressing an odorant receptor gene is influenced by sex and parenting status. *BMC Biology* **14** (2016).
